# Behaviour change interventions for the control and elimination of schistosomiasis: A systematic review of evidence from low- and middle-income countries

**DOI:** 10.1371/journal.pntd.0011315

**Published:** 2023-05-10

**Authors:** Carlos A. Torres-Vitolas, Suzan C. M. Trienekens, Willemijn Zaadnoordijk, Anouk N. Gouvras

**Affiliations:** 1 Unlimit Health, London, United Kingdom; 2 School of Public Health, Imperial College London, London, United Kingdom; 3 School of Biodiversity, One Health and Veterinary Medicine, College of Medical, Veterinary and Life Sciences, University of Glasgow, Glasgow, United Kingdom; 4 Ares Trading S.A. (a subsidiary of Merck KGaA, Darmstadt, Germany), Eysins, Switzerland; 5 Global Schistosomiasis Alliance, London, United Kingdom; Federal University of Ceará, Fortaleza, Brazil, BRAZIL

## Abstract

**Background:**

For the last two decades, schistosomiasis control efforts have focussed on preventive treatment. The disease, however, still affects over 200 million people worldwide. Behaviour change (BC) interventions can strengthen control by interrupting transmission through modifying exposure behaviour (water contact) or transmission practices (open urination/defaecation); or through fostering treatment seeking or acceptance. This review examines these interventions to assess their effectiveness in modifying risk practices and affecting epidemiological trends.

**Methodology/Principal findings:**

A systematic multi-database literature search (PROSPERO CRD42021252368) was conducted for peer-reviewed publications released at any time before June 2021 assessing BC interventions for schistosomiasis control in low- and middle-income countries. 2,593 unique abstracts were identified, 66 were assigned to full text review, and 32 met all inclusion criteria.

A typology of intervention models was outlined according to their use of behaviour change techniques and overarching rationale: health education (HEIs), social-environmental (SEIs), physical-environmental (PEIs), and incentives-centred interventions (ICIs). Available evidence does not allow to identify which BC approach is most effective in controlling risk behaviour to prevent schistosomiasis transmission. HEIs’ impacts were observed to be limited by structural considerations, like infrastructure underdevelopment, economic obligations, socio-cultural traditions, and the natural environment. SEIs may address those challenges through participatory planning and implementation activities, which enable social structures, like governance and norms, to support BC. Their effects, however, appear context-sensitive. The importance of infrastructure investments was highlighted by intervention models. To adequately support BC, however, they require users’ inputs and complementary services. Whilst ICIs reported positive impacts on treatment uptake, there are cost-effectiveness and sustainability concerns. Evaluation studies yielded limited evidence of independent epidemiological impacts from BC, due to limited use of suitable indicators and comparators. There was indicative evidence, however, that BC projects could sustain gains through treatment campaigns.

**Conclusions/Significance:**

There is a need for integrated interventions combining information provision, community-based planning, and infrastructure investments to support BC for schistosomiasis control. Programmes should carefully assess local conditions before implementation and consider that long-term support is likely needed. Available evidence indicates that BC interventions may contribute towards schistosomiasis control when accompanied by treatment activities. Further methodologically robust evidence is needed to ascertain the direct epidemiological benefits of BC.

## Author summary

Modifying people’s risk practices through behaviour change (BC) interventions can strengthen schistosomiasis control. Disease transmission can be interrupted by controlling peoples’ exposure to infected water, open defaecation practices, and by fostering treatment acceptance. We reviewed peer-reviewed publications released before June 2021 to assess the effectiveness of BC projects in decreasing risk practices and disease prevalence. 32 publications reporting on 31 projects based in Low- and Middle-Income countries were fully examined. Projects used four approaches: health education (HEIs), social-environmental (SEIs), physical-environmental (PEIs), and incentives-centred interventions (ICIs). Available data could not assert which approach was most effective in reducing risk behaviour. Structural barriers limited HEIs’ effects, whilst community social and material conditions affected those of SEIs. Both demanded comprehensive infrastructure investments. The cost-effectiveness of ICIs, in turn, remain unclear. Limited evidence of independent epidemiological impacts from BC was found. Indicative evidence, however, shows that BC projects could sustain gains attained through treatment activities. Overall, investment in integrated interventions appear needed to address exposure and transmission behaviour, whilst a context-driven strategic use of HEIs or SEIs appears useful to prompt treatment uptake. Despite BC’s potential, treatment activities appear essential for epidemiological effects.

## Introduction

Schistosomiasis, or bilharzia, is a parasitic infection caused by Schistosoma trematodes found in tropical and subtropical climates. Depending on the species, two types of disease are observed: urogenital (*S*. *haematobium*) or gastrointestinal schistosomiasis (*S*. *mansoni*, *S*. *japonicum*, *S*. *mekongi*, *S*. *guineensis*, *S*. *intercalatum*). The latter manifests through abdominal pain, diarrhoea, bloody stool, fever, and, in the long-term, it may cause enlarged spleen or liver, liver fibrosis, portal hypertension, or fluid accumulation in the peritoneal cavity [1,2]. Urogenital schistosomiasis typically manifests through haematuria (bloody urine) and dysuria (painful urination). Long-term complications may include fibrosis of the bladder and ureter, kidney damage, and bladder cancer [2,3]. Genital lesions, bleeding, pain and infertility may also be experienced [2,4]. There is growing evidence that urogenital schistosomiasis increases the risk of HIV infection [5,6]. In some cases, *S. mansoni* and *haematobium* can produce myelopathy, whereas *S. japonicum* encephalic disease [7,8]. Schistosomiasis is considered a ‘disease of poverty’, being endemic in regions lacking adequate water or sanitation infrastructure [9].

Despite continuous efforts by the international community to control the disease, schistosomiasis remains a global health concern. In 2012, the World Health Organization (WHO) agreed on a roadmap to eliminate schistosomiasis as a public health problem globally by 2025, defined as achieving a <1% prevalence of heavy-intensity infections among school-aged children (SAC) [10]. Whilst much progress has been made in reducing the prevalence of schistosomiasis, over 200 million people remain affected by the disease. Ninety percent of them live in Africa [11,12]. After a review of international progress combatting the disease, the WHO established 2030 as a new target for eliminating the disease as a public health problem by all endemic countries [11]. Preventive chemotherapy (PC) through mass drug administration (MDA) with praziquantel, investments in water and sanitation infrastructure, focal snail control with molluscicides, case management of infected individuals, as well as health education and behaviour change interventions constitute the main strategies recommended [11,13]. To date, however, MDA campaigns have been prioritised given their capacity to reduce disease prevalence in a cost-effective manner [12,13].

**Fig 1:**
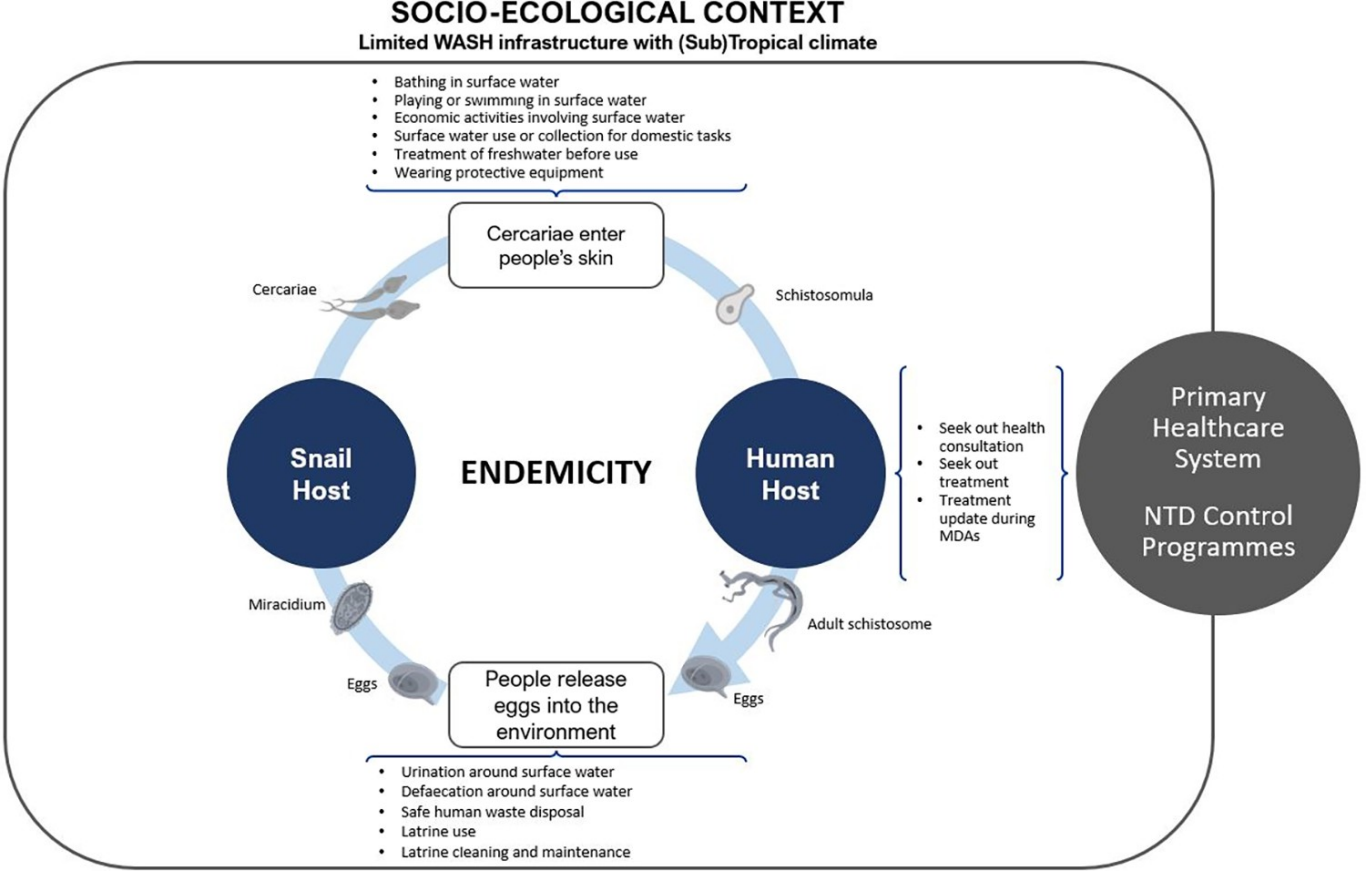
Human behaviour and the life cycle of schistosomiasis. Images of *Schistosoma* cercariae, miracidium, eggs and adult created by Servier Medical Art and available open access at: https://commons.wikimedia.org/wiki/File:Schistosoma_mansoni_cercaria.png, https://commons.wikimedia.org/wiki/File:Schistosoma_mansoni_miracidium_(01).png, https://commons.wikimedia.org/wiki/File:Schistosoma_mansoni_egg_(01).png, https://commons.wikimedia.org/wiki/File:Schistosoma_mansoni_female.png. All other images produced by the lead author.

Despite the less central role that behaviour change (BC) has played in ongoing efforts to control schistosomiasis, there is a growing view that modifications of people’s practices can help reducing the risk of transmission by disrupting the disease cycle [11,14,15]. Human behaviour is constitutive of the disease’s epidemiology. Humans contract the disease when they interact with infested water, allowing cercariae to enter their skin; once acting as hosts, people may avoid or reject treatment opportunities available at primary health centres or offered through MDAs, thus enabling schistosomulae to reach adulthood and produce eggs; which, finally, are released to the environment when people practice open urination and defaecation near fresh water sources, infecting intermediate snail hosts and continuing the cycle (See Fig 1). BC interventions may reduce or stop altogether the occurrence of such risk practices in various ways. They may convince people to adopt alternative practices (e.g., ground games instead of recreational swimming), promote the use of protective gear (e.g., gumboots or gloves), build water infrastructure, or encourage the use of treated water (e.g., heating, chlorination, filtration, or ultraviolet disinfection) [16–18]; improve hygiene and sanitation standards, by encouraging local investments in sanitation infrastructure or tackling the safety, privacy, and cost-benefit considerations that prevent their widespread use [19,20]; and encourage treatment seeking or MDA uptake by addressing the lack of knowledge on schistosomiasis or treatment, fears of side effects, negative rumours, or the perceived unimportance of the disease [21,22]. To date, however, there is no certainty concerning which type or combination of BC may be most efficient to control risk practices and schistosomiasis transmission [23]. In general, modelling studies and empirical assessments of persistent hotspots have highlighted that, given the potential high-rate of reinfection for the disease even when reaching the WHO’s recommended therapeutic coverage targets (>75% among SAC), further attention should be placed on the potential contributing role of modifying risk human behaviour for controlling schistosomiasis [24–26]. The most recent WHO’s programmatic guidelines, correspondingly, recommend that BC activities should be implemented alongside MDA as well as water hygiene and sanitation initiatives (WASH) [13].

To date, however, there has been no systematic examination of the capacity of existing BC approaches to control those risk practices necessary to interrupt the schistosomiasis cycle of transmission. The WHO acknowledges there is a critical need for strengthening the evidence concerning the effectiveness of BC interventions for fostering treatment compliance and healthy behaviours [11,13]. This study aims to fill this gap by conducting a systematic review of interventions conducted in Low- and Middle-Income countries that aimed to modify people’s behaviours relevant to schistosomiasis transmission. Specifically, this review will establish the type of BC strategies used in the sector and conduct a narrative synthesis of their reported effects on people’s risk behaviour as well as on reducing disease prevalence or intensity. Evidence-based lessons will be drawn to inform the design of future BC initiatives.

## Methods

### Eligibility

This review has two primary objectives. First, to determine the BC strategies or approaches currently being used for the prevention and control of schistosomiasis in Low- and Middle-Income Countries (LMIC) and, second, to assess the effectiveness of such strategies in positively altering the occurrence of exposure, transmission, or treatment uptake and seeking practices in communities or schools within LMIC. A secondary research question is to examine what evidence is currently available that indicates that changes in people’s risk practices can lead to significant improvements in the disease epidemiology in LMIC settings.

To address those queries, this review included peer-reviewed journal articles reporting on evaluations of BC interventions for schistosomiasis control in LMIC, following the classification by the Organisation for Economic Co-operation and Development [27]. This regional scope responded to the concentration of schistosomiasis infections and control efforts in those settings [11]. No restrictions were established concerning schistosomiasis species. All publications released prior to the search date, June 2021, were considered. Opinion and review pieces were excluded.

Behaviour change for schistosomiasis control was defined as any intervention that sought to modify people’s practices associated with the disease’s epidemiology. This included practices that put them at risk of acquiring the infection, or exposure behaviour (e.g., water contact); facilitate the spread of the disease, or transmission behaviour (e.g., open defaecation); or enable the uptake of or search for treatment (e.g., participation in MDAs). No restrictions were set concerning the strategies used to promote BC. The review included interventions targeting children, adults (>15), or community members in general. Interventions that solely reported on changes in knowledge or awareness were excluded since improvements in knowledge cannot be equated with BC [28]. BC interventions needed to explicitly aim to control schistosomiasis. Non-disease specific projects somewhat relevant to its epidemiology were excluded (e.g., generic hygiene and sanitation interventions).

Experimental and non-experimental evaluations were considered. This included observational studies, single-arm intervention studies, and randomised and non-randomised trials contrasting an intervention against a control group. Two types of outcome measures were judged acceptable. Direct indicators of BC, concerned with self-reported or observed changes in the frequency or proportion of beneficiaries’ exposure, transmission, or treatment seeking or uptake actions. Acceptable proxy outcome measures, next, comprised observed changes in schistosomiasis prevalence and observed or self-reported changes in sanitation infrastructure (e.g., number of latrines); treatments requested or used by targeted communities; ownership of relevant equipment (e.g., gumboots); and measurements of attitudes towards preventive measures.

This study’s protocol was registered at the International Prospective Register of Systematic Reviews (PROSPERO) (No: CRD42021252368).

### Search strategy

The search strategy drew on the Problem/Population, Intervention, Comparison, and Outcome (PICO) framework [29], albeit discarding the ‘comparison’ criterion since the use of control groups or comparators is not standard in the literature (Table 1). Search terms were agreed by all co-authors and trialled before implementation.

**Table 1:** Search terms.

Dimension	Terms	Connector
Problem	schistosom* OR bilharzia*	AND
Intervention	educati* OR sensitis* OR sensitiz* OR mobiliz* OR mobilis* OR informati* OR promoti* OR communicati* OR train* OR incentiv* OR infrastructure OR equipment OR latrine* OR sanitation	AND
Outcome	behavio$r* OR practice* OR coverage OR uptake OR compliance OR adherence OR attitud* OR participat* OR accept* OR resist* OR reject* OR avoid* OR hygien* OR wash* OR def$ecat* OR urinat* OR bath* OR swim* OR “water contact” OR “water-contact” OR “health-seeking” OR “health seeking” OR demand	-

A literature search of bibliographic databases EMBASE, SCOPUS, Web of Science, and PubMed / MEDLINE took place in June 2021. To ensure completeness, a manual search of publications was conducted by examining references from systematic reviews discussing MDAs [21,30–33], health education [23,34], and sanitation [17,35–38] strategies for schistosomiasis control as well as by requesting co-authors to identify studies considered valuable for this review.

**Fig 2:**
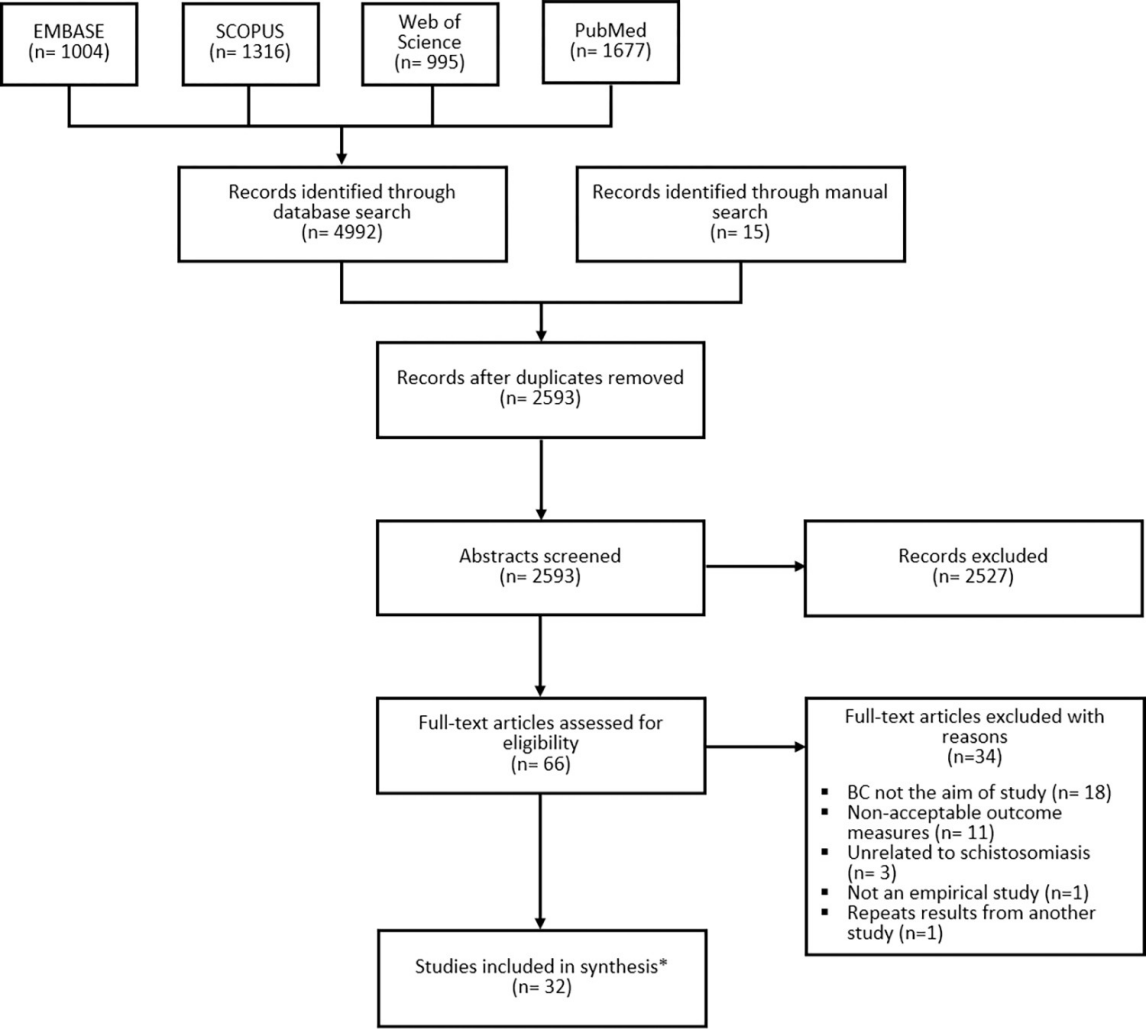
PRISMA chart. Two publications provided complementary information for a similar project. The total number of interventions reviewed is 31.

A total of 2,593 unique abstracts were obtained for abstract screening, of which 66 were selected for full-text review. The authors worked in pairs to independently conduct full-text reviews and agree on final inclusion. Thirty-two articles met all eligibility criteria (Fig 2). Two of them referred to the same intervention, henceforth yielding results for 31 projects. EPPI-Reviewer v.4 was used throughout the selection process [39].

### Quality assessment

The Joanna Briggs Institute’s Critical Appraisal tool for quasi-experimental evaluations, with an additional item on random assignation, was used to assess the studies’ quality [40] (See S1 Table). Authors worked in pairs to agree on final scores. No articles, however, were discarded since descriptive data in studies of ‘low’ methodological quality may still render valuable insights for a narrative review [41]. Quality assessments served the team to confirm if any claims of positive outcomes were backed up by studies considered methodologically robust and to what extent any methodological shortcomings could weaken such assertions. When relevant, the latter were highlighted in the text.

### Data extraction and synthesis

We conducted a framework synthesis to extract and synthesise the data from the selected publications [41]. First, descriptive information of BC interventions was extracted, including information about setting, targeted population, infection and behaviours, theoretical frameworks, intervention components, community participation, and education materials (See Table 2). Behaviour change techniques were identified for each intervention, following Michie et al.’s taxonomy [42] (See Table 3). Gathered information served to generate a typology of intervention models according to their overarching rationale, adapted from Mitchie et al.’s classification [43]. Second, extracted evaluation data, including evaluation design, methods, and outcome measures, served to assert the availability of evidence for either behavioural or epidemiological impacts (For full results, See S2 Table). Synthesised evidence of behavioural and epidemiological outcomes was contrasted according to the model of intervention to identify patterns in the data. A coding activity ensued to identify conditioning or mediating factors reported to explain final outcomes. Barriers were systematised according to the levels of analysis of the socioecological model of health behaviour [44]. Summaries of similarities, differences, and associations were produced for final analysis.

**Table 2:** Selected behaviour change interventions for schistosomiasis control according to intervention model.

Reference	Place	Setting	Time	Direct beneficiaries	Species	Behaviours targeted	Theory / Framework	Intervention components	Education materials	Community engagement
**HEALTH EDUCATION INTERVENTIONS**										
Chaula & Tarimo, 2014 [45]	Tanzania	Rural: Schools	2011–2012 ^a^	Schoolchildren (8–19)	*S*. *haematobium*	- Exposure - Treatment uptake	Not reported (N.R.)	- Health education: Advocacy campaigns alongside two successive MDA campaigns. - Treatment: MDA with praziquantel	N.R.	No
Cline & Hewlett, 1996 [46]	Cameroon	Rural: villages	1991–1993 ^a^	Schoolchildren ^a^	*S*. *haematobium*	- Exposure - Transmission - Treatment seeking	N.R.	- Health education: - Training for elementary school teachers and health centre staff. - Health centres developed education plans. - Private and public school, Koranic schools and community groups supported education activities. - Classroom-based education with sensitisation materials twice per year (‘season of transmission’ and ‘season of symptoms’). - Drawing and writing competitions, with prizes. - Supervision by health centre staff and district chief of preventive medicine. - Treatment: - School-based diagnostic tests and tests-and-treatment services in primary care at a cost (0.25$ and 1.50$, respectively). - Snail control: Molluscicides in two intervention villages.	- lip-charts^b^ - Posters^b^ - Brochures^b^	Community organisations supported dissemination of health messages.
Ejike et al., 2017 [47]	Nigeria	Rural: Schools	Jul–Aug 2014	Schoolchildren (5–19)	*S*. *haematobium*	- Exposure	N.R.	- Health education: Play with educational board game daily at school breaks for 2 months.	- Board game	No
Ejike et al., 2021 [48]	Nigeria	Rural: schools	Oct 2018-Mar 2019	Schoolchildren (5–19)	*S*. *haematobium*	- Exposure - Transmission - Treatment uptake	N.R.	- Health education: Playing with board game daily during school breaks, over 6 months. - Treatment: MDA with praziquantel	- Board game	No
Favre et al., 2021 [49]	Brazil	Urban and Rural: schools	Ag 2013 –Dec 2015	Schoolchildren (10–15)	*S*. *mansoni*	- Exposure - Treatment uptake	N.R.	- Health education: - 5-day training for schoolteachers with 6-month refresher session. - Twice a week lectures for schoolchildren over 3 months. - Thematic fairs (e.g., song, plays, games). - PC treatment: Targeted treatment with praziquantel	- Animated video ^c^ - Video-documentary ^c^ - Snail samples.	No
Garba et al, 2001 [50]	Niger	Rural: villages and schools	1991–1996	General population	*S*. *haematobium*	- Exposure	N.R.	- Health education—Community: - Informative video shown in villages (up to 5 times). - Community discussions led by local health and sanitation technicians using flipcharts. - Health education—Schools: - Projection of 26m informative video in local dialect. - Distribution of informative materials and schoolbooks.	- Educational video^b^ - Posters and flipcharts^b^ - School books^b^	No
Guang-Han et al., 2005 [51]	China	Rural: schools and villages	1992–1994 (2 villages) 1994–1996 (4 villages) ^a^	Schoolchildren (6–15) and adults (16–60)	*S*. *japonicum*	- Exposure - Treatment uptake	N.R.	- Health education: - Health information through videos and exhibitions of schistosomes and snails. - Informative sessions with separate tailored messages for men, women, and children. - Teachers praised pupils’ good behaviour and penalised incorrect practices. - Public information: Warning boards near high-transmission areas. - Treatment: MDA with praziquantel.	- Video^b^ - Schistosome and snail exhibition - Warning boards^b^	No
Hong et al., 2011 [52]	China	Rural: communities	2005–2008 ^a^	Schoolchildren (6–15) and adults (16–60)	*S*. *japonicum*	- Exposure - Treatment uptake	N.R.	- Health education: Health information through videos, cartoons, and booklets thrice a year over three years. - Infrastructure: Lavatories and tap water for all households. Public toilets built at landing sites. - Equipment: Containers for collection of faeces for fish folk. - Snail control: Twice a year surveys, with molluscicide treatment. - Treatment (humans): Targeted treatment with praziquantel. - Treatment (livestock): Annual targeted treatment of livestock.	- Cartoons ^b^ - Videos ^b^ - Comic-style booklets ^b^	No
Jia-Gang et al., 2005 [53]	China	Rural: villages	Feb. 1999 –Feb. 2000	Children and adults (6–60)	*S*. *japonicum*	- Treatment seeking	N.R.	- Health education: - Health information through videos and exhibitions of schistosome and snail samples. - Training on symptoms, prevention, and importance of early treatment with men, women, and children. - Treatment: MDAs with praziquantel for control group and self-referral for intervention group.	- Video ^b^ - Schistosome and snail exhibition	No
Lansdown et al., 2002 [54]	Tanzania	Rural: schools	Mar 1998 –Feb 1999	Schoolchildren (7–15)	*S*. *mansoni and*. *haematobium*	- Exposure - Transmission	N.R.	Health education:- Briefing to community leaders. - 4-day workshop for teachers on active teaching, parasitology, and prevention. - Classroom-based teaching on water use, prevention and sanitation. Locally developed materials: songs, poems, pictures and plays.	- Message boards ^**b**^ - Illustrations ^**b**^	- No
N’Diaye et al., 2016 [55]	Senegal	Rural: villages	2008–2015^a^	Children (0–14)	*S*. *mansoni* and *haematobium*	- Exposure - Transmission	N.R.	Health education:- Village meetings with video and card games and snail samples organised by civil and religious authorities alongside MDA campaigns. - Sensitisation focus on mothers with small children.Treatment: MDAs with praziquantel during 2009–14, targeted treatment in 2015 and for pre-SAC (0–5) in 2014.Infrastructure: Pit latrines, one per ten residents built over 6 years.	- Card games illustrating life cycle ^**b**^ - Video ^**b**^ - Snail samples	- Villagers’ input for latrine design. - Villagers helped building latrines.
Nagi et al. 2005 [56]	Yemen	Rural: village and school	Sep 1999 –Dec 2001	General population	*S*. *haematobium*	- Exposure - Treatment uptake	N.R.	Health education:- 2-day training sessions for teachers. - Trained teachers led weekly educational sessions at school. - Weekly sessions for non-enrolled children and pre-school children with their parents. - Educational sessions during khat sessions and Friday prayers. - Treatment: MDA with praziquantel.	- Posters ^**b**^	No
Oyeyemi et al., 2018 [57]	Nigeria	Rural: communities	Jan 2016	General population	*S*. *haematobium*	- Exposure	N.R.	- Health education: Disease information provided during urine sample collection. Infrastructure: Community borehole (post-sample collection). Treatment: Targeted treatment with praziquantel.	N.R.	No
Stothard et al., 2016 [58]	Tanzania	Urban and rural: schools	Dec 2005 –Jan 2007	Schoolchildren (Primary—Class V)	*S*. *haematobium*	- Exposure. - Transmission	N.R.	- Health education: - 30-minute talk by health educator prior distribution of comic strip. - Comic book integrated into the health curricula over a year.	*Juma na kichocho* comic strip [59]	No
Wang et al., 2013 [60]	China	Rural: villages	April to June 2009	Adults	*S*. *japonicum*	- Exposure - Transmission - Treatment uptake	N.R.	- Health education: - Class-based sessions with posters, display boards, and a video. - Reinforcement sessions with prize-winning quizzes. - Distribution of small goods with printed information to residents. - Treatment: Targeted treatment with praziquantel.	- Display boards [60] - Video ^**b**^ - Pamphlets ^**b**^ - Utensils: towels, bags, containers [60]	No.
Wolmarans & de Knock, 2009 [61]	South Africa	Rural: schools	Jan 2004—Dec 2006.	Schoolchildren (4–14)	*S*. *haematobium*	- Exposure	N.R.	- Health education: Puppet show delivered over a 2-year period. - Treatment: Targeted treatment with praziquantel.	- Puppet show ^**b**^	No.
Yuan et al., 2000 [62]	China	Rural: schools	July 1996	Schoolchildren (4^th^ grade)	*S*. *japonicum*	- Exposure	N.R.	- Health education: - 15-minute animation video presented in class - 10-minute discussion with local NTD control staff. - Distribution of educational comic to pupils.	- Animated video ^**b**^ - Comic book ^**b**^	No.
**SOCIAL ENVIRONMENTAL INTERVENTIONS**										
Nsowah-Nuamah et al. 2001 [63]	Ghana	Rural: villages	Ap. 1994- May 1997	General population	*S*. *haematobium*	- Exposure - Transmission	N.R.	- Health education—Passive: - Video shown in the community (once). - Briefing to local leaders with posters and flipcharts. - Educational sessions led by teachers, village, and religious leaders during regular meeting times of their organisations. - Health education—Active: - Informative video shows once a month, per 18 months. - In-depth training for community volunteers. - Trained volunteers visited parents of SAC twice a week for 18 months to educate on the disease and control measures. - Spelling and quiz competitions. c. Community organisation—Active: - Community meetings to plan activities. - Committees for safe water, latrine building and environmental management. d. Infrastructure: - Financial and technical assistance for construction of safe water supply and pit latrines as well as weed removal. - Assistance available to all communities upon request to local public development agencies. e. Treatment: MDA with praziquantel	- Posters - Flipcharts - Videos	- Village committees organised. - Labour for infrastructure. - Provision of local building materials (e.g., sand).
Hurlimann et al., 2018 [64]	Cote D’Ivoire	Rural: communities	Aug. 2011—Aug. 2012	General population	*S*. *mansoni and haematobium*	- Transmission	Community-led total sanitation (CLTS) [65]	- Planning: - Workshop with local leaders on the intervention to foster buy-in. - Local health authorities, town-hall officials, health and demographic surveillance officers and other stakeholders trained on CLTS. - Village meetings with participatory assessment of sanitation conditions and mapping of faecal contamination (trigger disgust). - Villagers design and agree on plans to become open-defaecation free (ODF). - Public event to present action plans. - Technical assistance: Trained facilitators assisted and monitored latrine building. - Infrastructure: Latrine building. - Health education: - Participatory exercises assessed knowledge and education needs. - Meetings to discuss and inform about the disease and risk practices. - Treatment: MDA with albendazole, praziquantel and ivermectin.	- Booklets ^b^ - Posters ^b^ - Pictures ^b^	- Examination and mapping of open defaecation problems. - Outline of prevention measures and timelines. - Assessment of education needs and plans for education activities. - Labour for latrine building. - Local leaders led implementation.
Madon et al., 2018 [66]	Tanzania	Rural: communities	Nov. 2015 –Ap. 2016	General population	*S*. *mansoni and haematobium*	- Transmission	Enhanced Development Governance (ad-hoc framework)	- Organisational support: Expanded membership to social services committees (SSCs), incorporating village council members, health workers, community bank, youth & religious groups, teachers, and drug distributors. - Financial support: Start-up funds and training for income-generating activities and NTD / sanitation initiatives: cleaning schools, clearing garbage, building wells and latrines. - Health education: - NTD-WASH education through lectures and discussions. - Health education sessions held in individual households and public spaces. - Technical support: Training on design and management of NTD projects.	N.R.	- Village council members identified new SSC members. - SSCs organised monthly village clean-up operations. - SSCs agreed on penalties to ensure compliance with sanitation standards.
Mwanga et al., 2013 [67]	Tanzania	Rural: communities ^d^	2008 ^a^	General population	*S*. *mansoni*	- Exposure. - Treatment seeking	Participatory Hygiene and Sanitation Transformation (PHAST) [68,69]	- Health education: Trained community facilitators organised meetings with up to 24 adults to discuss risk practices and transmission in the community. - Planning: Villagers outlined community objectives, plans of action, goals, and monitoring activities. - Treatment: MDA with praziquantel.	N.R.	- Participatory diagnosis - Participatory planning - Participatory monitoring and evaluation ^**e**^
Mwanga et al., 2015 [70]	Tanzania	Rural: communities ^d^	2009^a^	General population	*S*. *mansoni*	- Exposure.	Participatory Hygiene and Sanitation Transformation (PHAST) [68,69]	- Health education: Village meetings discussed risk practices and transmission in the community context. - Planning: Villagers outlined community objectives and plans of action. - Treatment: MDA with praziquantel and Albendazole. - Infrastructure: Pumped well.	N.R.	- Participatory diagnosis - Participatory planning - Participatory monitoring and evaluation ^**e**^
Person et al., 2021 [71] Knopp et al., 2019 [72]	Tanzania	Rural and Urban: schools	2011–2017^a^	Children (9–12)	*S*. *haematobium*	- Exposure - Transmission - Treatment uptake	Human-Centred Design [73]	- Health education: - Classroom based teaching with interactive materials and activities. - Public ‘Kichocho day’ events including dramas, poems, and safe games with participation of the general population. - Village meetings with educational films. - Infrastructure: Urinals and washing platforms built following residents’ inputs. - Treatment: Biannual MDAs with praziquantel.	- lipchart with pictures of transmission sites, snails, risk activities, and treatment ^b^ - Blood fluke pictures ^b^ - Snail boards ^b^ - Drawings of lifecycle ^b^	- Teachers co-designed education materials, games, and plays. - Residents selected and co-designed BC strategies. - Residents co-designed and built urinals and washing platforms.
Rassi et al., 2019 [74]	Mozambique	Rural and Urban: communities	Aug. 2014—Sep 2015,	General population	*S*. *haematobium*	- Exposure - Transmission - Treatment uptake.	Community Dialogue Approach(75)	- Health education: - Training for volunteers on the disease, prevention, and facilitation. - Volunteers led community meetings (n = 1,500). - Meetings informed on causes, transmission, and prevention. - Planning: Residents proposed and agreed on locally-led control measures, including commitments to treatment uptake, exposure reduction, and sanitation initiatives. - Treatment: MDA with praziquantel	- lipchart illustrating intervention ^**b**^	- Local volunteers organised community dialogue events. - Participatory design of solutions and measures to promote control activities.
**PHYSICAL ENVIRONMENTAL INTERVENTIONS**										
El Kholy et al., 1989 [76]	Kenya	Rural: villages	1984–1985	General population	*S*. *haematobium*	- Exposure	N.R.	a. Infrastructure: Boreholes built, b. Treatment: MDA with praziquantel for SAC	Not Applicable (N.A.)	N.R.
Freeman et al., 2013 [77]	Kenya	Schools ^f^	May 2007—Nov. 2008	Schoolchildren (7–13).	*S*. *mansoni*	- Exposure - Transmission: latrine use.	N.R.	- Training: One parent and one teacher per school trained on health, hygiene, and maintenance of infrastructure. - Infrastructure: Latrines built according to school size (4–7 / school). - Equipment: Provision of hand washing and drinking water containers. - Technical support: One-year supply of point-of-use water treatment product. - Treatment: MDA with Albendazole and targeted treatment with praziquantel.	N.R.	No.
Kosinski, et al., 2016 [78]	Ghana	Rural: communities	Jun. 2008-Ag. 2010	Schoolchildren (8–22)	*S*. *haematobium*	- Exposure: water-contact.	N.R.	- Infrastructure: 30 m^2^ concrete pool fed by rainwater and pumped groundwater. - Treatment: MDA with praziquantel.	N.A.	No
Noda et al., 1997 [79]	Kenya	Rural: village	Feb. 1984	General population	*S*. *haematobium*	- Exposure: water-contact.	N.R.	- Infrastructure: - One free of charge shower unit with five rooms - Five community standpipes installed, payment per bucket	N.A.	- Water committees maintained infrastructure.
Wepne et al., 2019 [80]	Cameroon	Rural: village	2014–2017^a^	Adults: pregnant women	*S*. *haematobium*	- Exposure: water-contact.	N.R.	- Infrastructure: Installed community piped water, immediate payment upon use.	N.A.	No
**INCENTIVES CENTRED INTERVENTIONS**										
Fink & Rockers, 2017 [81]	Zambia	Urban and Rural: communities	July 2011	Early school aged children (6–7)	N. R.	- Treatment seeking / uptake: bring children to health centre.	Conditional cash transfer	- Financial support: Offers of cash payments if children brought to a health centre for check-up within 7 days.	N.A.	No
Muhumuza et al., 2014 [82]	Uganda	Rural: schools	May–July 2013	Schoolchildren (7–16)	*S*. *mansoni*	- Treatment seeking / uptake: MDA uptake.	N. R.	- Health education: 30-minute classes by trained teachers for two months to control and intervention groups. - Pre-treatment snacks: mango juice and doughnuts given prior drug distribution. - Treatment: MDA with praziquantel.	N.R.	No

a. No further details provided

b. Source material not referenced or reproduced

c. Videos available online: animated video, video-documentary

d. Study sites in different regions for publications by the same author

e. Items obtained from framework guidelines

f. Rurality not reported

## Results

### Geographical distribution

**Fig 3:**
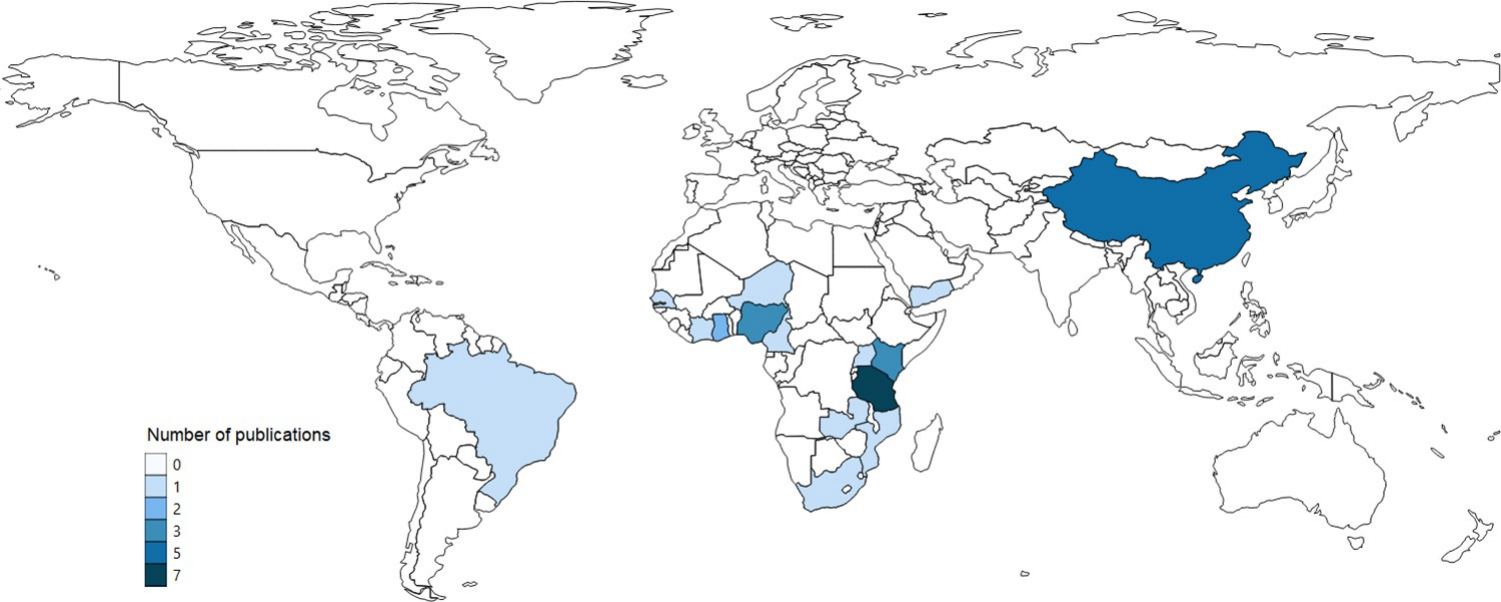
Geographical distribution of selected BC studies in LMIC countries. Vector and raster map data made available by Natural Earth and obtained open access from: https://www.naturalearthdata.com/downloads/110m-cultural-vectors/110m-admin-0-countries/.

The geographical distribution of BC interventions for schistosomiasis control was limited to 15 countries (Fig 3). Most of the projects were based in Africa (24 of 31), with most information coming from Tanzania (n = 7). Outside Africa, most evidence came from China (n = 5). Projects predominantly intervened in rural areas exclusively (n = 25).

### Quality assessment

The mean quality assessment score for all 32 publications was 6.2, on a 10-point scale. Sixteen publications scored ≥7, indicating an adequate body of evidence. Three methodological concerns were noted, including: not using behavioural indicators to assess outcomes (n = 5) [52,55,57,61,77], relying exclusively on ‘self-reported’ measures to assess BC (n = 14) [45,47,48,50,51,56,58,60,70,71,74,76,80,82], and lacking control groups in evaluation designs(n = 10) [52,55–58,67,70,74,79,80].

**Table 3:** Types of BC techniques used by selected projects according to intervention model.

Projects	Types of Behaviour Change Strategies									
	Knowledge	Consequences	Comparison of outcomes	Associations	Goals and Planning	Feedback and monitoring	Self-belief	Context	Reward and incentives	Scheduled consequences
	- Info. on transmission cycle and risk practices. - Instructions to adopt safe practices.	- Info. about health consequences. - Info. about social consequences - Info. about severity of consequences. - Info. about benefits.	- Credible actors explain pros and cons of behaviours. - Explicit comparison of prod and cons.	- Environmental reminders / stimuli for safe behaviour. - Environmental reminders of benefits.	- Analysis of problems and outline of correcting measures. - Planning how to implement measures. - Agreement on goals. - Public commitment to measures or goals.	- Monitoring of compliance. - Feedback to actors on performance.	- Advise or statements stressing local capacity to implement recommendations.	- Changes to the physical environment. - Changes to the social environment.	- Rewards or promises of rewards if progress towards compliance (material or immaterial).	- Arranged system of sanctions based on behavioural outcome.
**HEALTH EDUCATION INTERVENTIONS**										
Chaula & Tarimo, 2014 [45]	**X**	**X**								
Cline & Hewlett., 1996 [40]	**X**	**X**	**X**	**X**					**X**	
Ejike et al. 2017 [47]	**X**	**X**		**X**					**X**	
Ejike et al. 2021 [48]	**X**	**X**		**X**					**X**	
Favre et al., 2021 [49]	**X**	**X**	**X**	**X**						
Garba et al, 2001 [50]	**X**	**X**	**X**							
Guang-Han et al., 2005 [51]	**X**	**X**	**X**	**X**					**X**	**X**
Hong et al., 2011 [52]	**X**	**X**						**X** ^**a**^		
Jia-Gang et al., 2005 [53]	**X**	**X**								
Lansdown et al., 2002 [49]	**X**	**X**	**X**	**X**						
Nagi et al. 2005 [56]	**X**	**X**	**X**							
N’Diaye et al., 2016 [55]	**X**	**X**						**X** ^**a**^		
Oyeyemi et al., 2018 [57]	**X**	**X**						**X** ^**a**^		
Stothard et al., 2016 [58]	**X**	**X**	**X**	**X**						
Wang et al., 2013 [60]	**X**	**X**		**X**					**X**	
Wolmarans & de Knock, 2009 [61]	**X**	**X**								
Yuan et al., 2000 [62]	**X**	**X**								
**SOCIAL ENVIRONMENTAL INTERVENTIONS**										
Nsowah-Nuamah et al. 2001 [63]	**X**	**X**		**X**	**X**			**X** ^**a**^^,^^**b**^	**X**	
Hurlimann et al., 2018 [64]	**X**	**X**			**X**	**X**	**X**	**X** ^**b**^		
Madon et al., 2018 [66]	**X**	**X**			**X**	**X**	**X**	**X** ^**a**^^,^^**b**^		**X**
Mwanga et al., 2013 [67]	**X**	**X**			**X**	**X**	**X**	**X** ^**b**^		
Mwanga et al., 2015 [70]	**X**	**X**			**X**	**X**	**X**	**X** ^**a**^^,^^**b**^		
Person et al., 2021 [71] Knopp et al., 2019 [72]	**X**	**X**	**X**	**X**	**X**		**X**	**X** ^**a**^^,^^**b**^		
Rassi et al., 2019 [74]	**X**	**X**			**X**		**X**	**X** ^**b**^		
**PHYSICAL ENVIRONMENTAL INTERVENTIONS**										
El Kholy et al., 1989 [76]								**X** ^**a**^		
Freeman et al., 2013 [77]	**X**							**X** ^**a**^		
Kosinski, et al., 2016 [78]								**X** ^**a**^		
Noda et al., 1997 [79]								**X** ^**a**^		
Wepne et al., 2019 [80]								**X** ^**a**^		
**INCENTIVES BASED INTERVENTIONS**										
Fink & Rockers, 2017 [81]									**X**	
Muhumuza et al., 2014 [82]									**X**	

a. Physical environment

b. Social environment

### Key design characteristics

**Fig 4:**
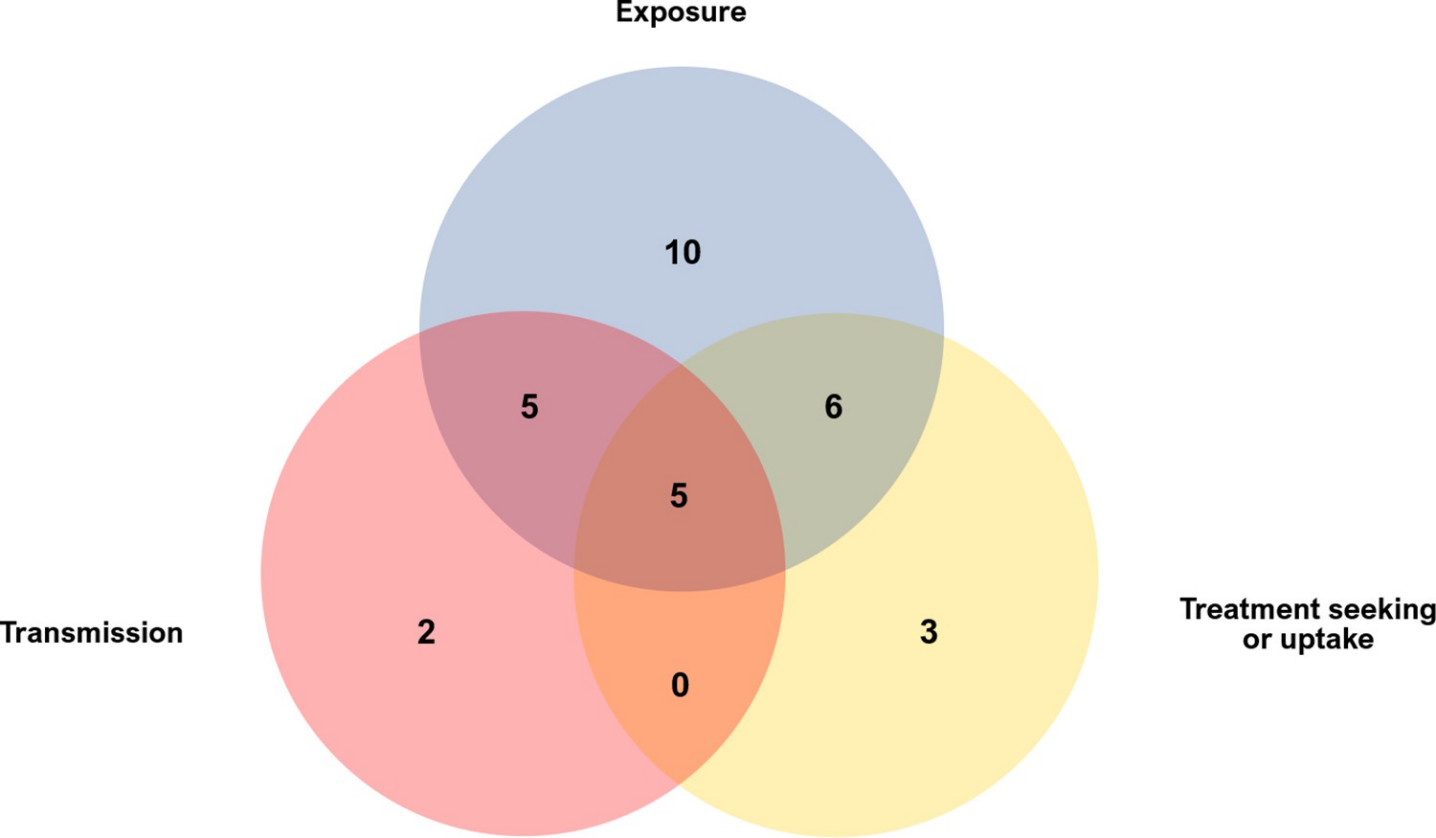
Behaviours targeted by BC interventions for schistosomiasis control in LMIC.

The behavioural focus of the interventions varied across exposure (n = 26), transmission (n = 17) and treatment seeking/uptake (n = 19). More than half of the projects aimed to modify two or more behaviours simultaneously (n = 16) (Fig 4). Overall, exposure and treatment seeking or uptake behaviour were the most commonly targeted for schistosomiasis control.

Empirical approaches apparently guided the design of most interventions. Three fourths of the projects (24 of 31) did not report using a theoretical framework, either psychological or educational. The remaining seven used six different approaches: conditional cash transfers [81], community-led total sanitation (CLTS) [64], human-centred design [71,72], enhanced development governance [66], participatory hygiene and sanitation transformation (PHAST) [67,70], and community dialogue approach [74].

Four intervention models were identified according to the BC techniques used (See Table 3): (1) Health education, (2) social-environmental, (3) physical-environmental, and (4) incentives-centred interventions. Health education interventions (HEIs) were the most common (n = 17). It concerned projects that aimed to drive BC through individual-level improvements in knowledge of schistosomiasis [45–52,54–58,60–62,83]. HEIs mostly relied on information provision techniques, communicating messages about the contextual factors driving exposure and transmission, negative health and socio-economic consequences of contracting the disease (and their severity), and the positive impacts of adopting preventive practices or accepting treatment. Some projects strengthened those activities by integrating BC instructions into school-classes and mobilising teachers (credible sources) [46,49,49–51,54,56,56,58,58]. Other techniques played a secondary role (e.g., rewards).

Social-environmental interventions (SEIs, n = 7) expected to achieve BC by reshaping beneficiaries’ social environment, ensuring that community-level formal and informal norms and structures (e.g., governance) were aligned to enable or enforce adopting recommended practices [63,64,66,67,70–72,74]. They customarily used participatory community-based frameworks (e.g., CLTS or PHAST), supplementing educational BC components with goal-setting, planning, monitoring, and social-restructuring strategies with the active involvement of residents. Activities often included joint assessments of risk behaviours and living conditions, developing operational plans, establishing targets and timelines, public or formal commitment to goals, and adopting supervisory mechanisms to monitor compliance and progress. Community leadership across all implementation stages was expected to enhance perceived self-capacity (self-efficacy).

Physical environmental interventions (PEIs), focussed on modifying the beneficiaries’ material surroundings through infrastructure or equipment investments [76–80]. Incentives-centred interventions (ICIs), concerned the provision of material resources conditioned on following recommended practices: cash, if parents took children to a health centre [81] and food, if children participated in MDA activities [82].

### Effectiveness of health education interventions

Seventeen HEIs were assessed. Most attempted to modify two or more risk practices simultaneously (n = 11). Three targeted all three risk practices [46,48,60]. Four did not report behavioural indicators so their effectiveness could not be assessed [52,55,57,61]. Results varied according to the type of BC promoted. HEIs mainly reported mixed [47,48,50,51,62] or negative results [45,49,58,60] for exposure behaviour. In contrast, all bar one intervention on treatment seeking or uptake reported favourable outcomes [46,47,49,51,53,56,60]. On transmission, two HEIs reported positive outcomes [58,60] and two mixed results [48,54].

One HEI reported comprehensive positive outcomes for exposure. It sensitised children through trained school and religious teachers during school classes, Khat (a plant that is chewed) sessions, and Friday prayers for 2 years in Yemen [56]. Results indicated a drastic reduction in surface water contact (from 98% to 3.6%) and adoption of preventive measures (from 0% to 88%). The project lacked a control group and relied exclusively on self-reported measures so results should be seen with caution. Project activities reportedly helped achieve >95% coverage during a first MDA campaign.

Three similar China-based projects, in turn, conducted short-term educational activities (<1 year) involving class-based sessions, sample exhibitions (snails and schistosomes), and audio-visual and printed materials with sensitisation messages (e.g., domestic goods). One, in Jiangxi province, targeted exposure practices and treatment uptake and reinforced sensitisation through teachers praising good behaviour and reprimanding noncompliance [51]. Another, in Sichuan province, targeted all three risk practices and reinforced learning through prize-winning quizzes [60]. The third intervention, also in Jiangxi province, taught residents about symptoms and the importance of early treatment to encourage self-referring for treatment to local medical teams (“passive chemotherapy”) [53]. The first project reported a significant decrease in unsafe water contact among children and women but not among men, whilst the second found no significant changes in adults’ unprotected contact with surface water. All three interventions reported positive outcomes for treatment uptake. The first one in Jiangxi province reported improvements in treatment uptake and attitudes for all groups, marginal improvements in adults’ attitudes were found in Sichuan, whilst the ‘passive chemotherapy’ project reported that 90% of infected residents self-referred for treatment. In Sichuan, a marginally significant reduction in adults’ open defaecating was found.

Three school-based HEIs with similar approaches provided teacher training, integrated schistosomiasis-related content into the curricula, and fostered learning through ludic activities (e.g., songs, poems, and plays) and public events (e.g., thematic fairs). One, in Cameroon, developed the teaching plan with local health centres, lasted an academic year, and provided low-cost diagnostic and treatment services [46]. Another, in Brazil, supported learning activities with audio-visual materials for three months, and provided free-of-charge test-and-treat services [49]. These projects reported an increased demand for schistosomiasis consultations in health centres and greater adherence to test-and-treat activities, respectively. In Brazil, however, no significant changes concerning risk water contact activities were found. The third project, in Tanzania, lasted an academic year, and targeted exposure and transmission practices [54]. It reported proxy measures, showing that intervened schools were more likely to offer treated water and keep good latrine hygiene, but no differences were observed concerning open defaecation.

Game-based learning including different versions of the boardgame “Schisto and Ladders”, containing health education information, was used to modify schoolchildren’s water contact behaviour, first, and all three risk practices subsequently [47,48]. The game was played during classes for two and six months respectively. The earlier project reported a significant decrease in schoolchildren’s daily exposure to dam-water but no changes in playing activities. The second (improved) version, was associated with self-reported improvements in wearing rubber boots, washing, water fetching, and bathing but not for fishing. On transmission, urination in the river decreased but open defaecation remained unchanged. Also, 65% of schoolchildren who had previously rejected treatment registered for treatment post-intervention.

Two additional HEIs using non-traditional methods used an educational comic strip in primary school classes for a year in Zanzibar [58] and 15-minute animated videos, alongside comic strips and short discussions, among 4^th^ grade students in China [62]. The latter found that self-reported frequency of swimming did not change post-intervention, but self-reported and observed use of unsafe water sources decreased, although contact remained high (31.3%). The Zanzibar HEI found no changes in schoolchildren’s self-reported water contact but a significant decrease in open urination practices.

Two basic HEIs were conducted in Niger and Tanzania. The first one projected videos in villages and schools, followed by discussions, and distributed informative printed materials sporadically for 5 years [50], whilst the second provided information on transmission, prevention, and treatment during school-based MDAs over two annual campaigns [45]. Despite improvements in preventive practices, the Niger intervention found that two-thirds of participants continued exposure risk behaviour. In Tanzania, no significant improvements were found pertaining schoolchildren’s water contact practices and MDA coverage rates were low (<50%).

HEIs highlighted structural barriers conditioning their capacity to generate BC, including the lack of safe water and sanitation infrastructure [45,47–50,58,60,62]; rural occupations, exposing people to surface water and keeping them away from latrine installations [45,51,52,55,58,60]; and, socio-cultural traditions, like gender-based norms and traditional games [45,51,58]. The natural environment was likewise observed to condition the implementation of solutions (e.g., flooding makes exposure more likely) [51,58,60,62]. Inter-personal relations were seen to facilitate information sharing but also counteract BC, such as children being unable to alter adults’ views on domestic obligations (e.g., washing) [47,48,50,54,61]. Community engagement was seen useful mainly to add weight to health messages and mobilise resources to support dissemination [46,53,54].

### Effectiveness of social environmental interventions

Seven SEIs were reviewed, three targeted single behaviours [64,66,70]. Two attempted to control all three risk practices [71,74]. Most consistent positive outcomes were observed for those SEIs with a single focus on open defaecation [64,66]. Mixed or unfavourable results were mostly observed for exposure and treatment uptake practices (See Table 4).

**Table 4:** Summary of evaluation results for BC interventions. To access full results, see S2 Table.

REFERENCE	EVALUATION		BEHAVIOURAL OUTCOMES						CONDITIONING FACTORS					QUALITY APPRAISAL SCORE (Over 10)
	Design	Data sources	EXPOSURE		TRANSMISSION		TRATMENT SEEKING OR UPTAKE		Individual	Interpersonal	Community	Societal	Others	
			Significant beneficial change ^a^	No significant change ^b^	Significant beneficial change ^a^	No significant change ^b^	Significant beneficial change ^a^	No significant change ^b^						
**HEALTH EDUCATION INTERVENTIONS**														
Chaula & Tarimo, 2014 [45]	- Post measures - Non-equivalent control - Random assignation	- KAP survey		- Generic ^c^ - Domestic				- MDA uptake	- Occupation - Fear of side effects		- WASH infrastructure	- Socio-cultural traditions	- Long-term process	2
Cline & Hewlett, 1996 [46]	- Pre-post measures - Non-equivalent control - Non-random assignation	- Treatment records					- Treatment seeking				- Community engagement - Population dispersion	- Public programmes engagement - Health staff training		5
Ejike et al. 2017 [47]	- Pre-post measures - Equivalent control - Random assignation	- KAP survey	- Generic ^c^	- Playing						- Domestic relations - Social networks	- WASH infrastructure			8
Ejike et al. 2021 [48]	- Pre-post measures - Equivalent control. - Random assignation.	- KAP survey	- Rubber boots - Washing - Water fetching - Bathing	- Fishing	- Open urination (river)	- Defaecation (river)	- Treatment seeking			- Domestic relations	- WASH infrastructure			7
Favre et al., 2021 [49]	- Pre-post measures - Equivalent control - Random assignation	- KAP survey		- Generic ^c^			- Treatment seeking (overall)	- Treatment seeking (positive cases)	- Fear of side effects		- WASH Infrastructure - Population dispersion			8
Garba et al., 2001 [50]	- Post measures - Non-equivalent control - Non-random assignation	- KAP survey	- Prevention - Bathing (home)	- Bathing (surface water sources)					- Education	- Social networks	- WASH infrastructure			4
Guang-Han et al., 2005 [51]	- Pre-post measures - Equivalent control - Random assignation	- KAP survey	- Generic (women and children) ^c^	Generic (men) ^c^			- MDA uptake - Treatment attitudes		- Age - Sex - Occupation - Fear of side effects	- Teachers’ influence		- Gender - Socio-cultural traditions - Natural environment		6
Hong et al., 2011 [52]	- Pre-post measures - Single group - Non-random assignation.	- No behavioural measures	n.r.	n.r.			n.r.	n.r.	- Occupation		- Seasonal migration			3
Jia-Gang et al., 2005 [53]	- Pre-post measures - Non-equivalent control - Non-random assignation	- Treatment records					- Treatment seeking				- Community resources - History of public health interventions. - Community governance	- Strength of primary care system. - Public programmes engagement		8
Lansdown et al 2002 [54]	- Pre-post measures - Non-equivalent control - Random assignation.	- Direct observation	- Safe water provision		- Open defaecation	- Latrine hygiene - Open urinationd				- Domestic relations - Teacher’s influence	- Community resources	- Cooperation across public sectors. - Public programmes engagement.		7
N’Diaye et al., 2016 [55]	- Pre-post measures - Single group - Non- random assignation.	- No behavioural measures	n.r.	n.r.	n.r.	n.r.			- Occupation		- WASH infrastructure - Additional public infrastructure	- Nat. environment		2
Nagi et al. 2005 [56]	- Pre-post measures - Single group - Non-random allocation	- KAP survey	- Generic ^c^ - Prevention				- Treatment uptake		- School enrolment					3
Oyeyemi et al. 2018 [57]	- Pre-post measures - Single group - Non-random assignation	- No behavioural measures	n.r.	n.r.							- WASH infrastructure			3
Stothard et al., 2016 [58]	- Pre-post measures - Single group - Non-random assignation	- KAP survey		- Generic ^c^	- Open urination (river)				- Occupation		- WASH infrastructure	- Socio-cultural traditions - Natural environment		5
Wang et al., 2013 [60]	- Pre-post measures - Equivalent control - Random assignation	- KAP survey		- Generic - Protected contact	- Open defaecation (fields)		- Treatment attitudes		- Occupation		- WASH infrastructure	- Natural environment		9
Wolmarans & de Knock, 2009 [55]	- Pre-post measures - Equivalent control - Non-random assignation	- No behavioural measures	n.r.	n.r.						- Social networks				6
Yuan et al. 2000 [62]	- Pre-post measures - Equivalent groups - Random assignation	- KAP survey - Direct observation	- Safe water use	- Swimming - Unsafe water use							- WASH infrastructure	- Natural environment	- Short-term intervention	6
**SOCIAL ENVIRONMENTAL INTERVENTIONS**														
Nsowah-Nuamah et al. 2001 [63]	- Pre-post measures - Non-equivalent control - Non-random assignation	- Direct observation	- Safe water availability ^d^		- Latrines availability ^e^				- Age - Sex - Occupation		- Community resources - Community governance - Additional public infrastructure	- Nat. environment		7
Hurlimann et al., 2018 [64]	- Pre-post measures - Non-equivalent control - Random assignation.	- KAP survey - Direct observation			- Latrine use - Toilet use - Open defaecation				- Age - Sex - Occupation		- Social cohesion - Additional public Infrastructure	- Gender norms - Socio-cultural traditions	- Long-term process	8
Madon et al., 2018 [66]	- Pre-post measures - Equivalent control - Random assignation	- KAP survey - Direct observation			- Open defaecation - Latrine use - Latrine hygiene						- Community governance - Community resources	- Public programmes’ engagement	- Long-term process	7
Mwanga and Lwambo., 2013 [67]	- Pre-post measures - Single group - Non-random assignation	- KAP survey - Direct observation	- Generic ^c^ (children) - Water fetching - Bathing (women)	- Generic ^c^ (adults) - Bathing (men)			- Treatment seeking (women)	- Treatment seeking (men)	- Age - Sex		- WASH infrastructure	- Gender norms		5
Mwanga et al., 2015 [70]	- Pre-post measures - Single group - Non-random assignation	- KAP survey	- Generic ^c^ - Bathing						- Sex		- WASH infrastructure	- Gender norms		4
Person et al., 2021 [71]	- Post measures - Equivalent control. - Random assignation.	- KAP survey	- Domestic - Bathing	- Swimming / Playing			- Treatment rejection			- Adults’ influence	- WASH infrastructure	- Socio-cultural traditions		7
Knopp et al., 2019 [72]	- Pre-post measures - Equivalent control. - Random assignation.	- Treatment records						- Treatment uptake			- WASH infrastructure		- Long-term process	9
Rassi et al., 2019 [74]	- Pre-post measures - Single group - Non-random assignation	- KAP survey	- Swimming	- Bathing		- Latrine Use	- Treatment Uptake	- Treatment attitudes	- Occupation		- Community resources - WASH infrastructure	- Socio-cultural traditions	- Long-term process	6
**PHYISICAL ENVIRONMENTAL INTERVENTIONS**														
El Kholy et al, 1989 [76]	- Post measures - Non-equivalent control. - Non-random assignation	- KAP survey	- Drinking / Cooking - Bathing - Washing dishes	- Washing clothes							- Population dispersion - WASH infrastructure. - Additional public infrastructure	- Gender norms - Payment system - Natural environment		8
Freeman et al., 2013 [77]	- Pre-post measures - Equivalent control - Random assignation	- Direct observation	- Treated water availability ^d^	- Safe water provision ^d^	- Latrine availability ^e^						- Schools’ organisational capacity.	- Natural environment - Programme’s efficiency		8
Kosinski, et al., 2016 [78]	- Post measures - Single group - Non-random assignation	- Direct observation	- Generic ^c^						n.r.	n.r.	n.r.	n.r.	n.r.	6
Wepnje et al., 2019 [80]	- Pre-post measures - Single group - Non-random assignation	- KAP survey	- Generic ^c^	- Domestic - Bathing					- Education	- Marital status	- Population dispersion - Additional public infrastructure.	- Gender norms - Payment system		7
Noda et al., 1997 [79]	- Pre-post measures - Single group - Non-random assignation	- Direct observation - Standpipe use records	- Generic ^c^ (total) - Washing clothes (river) - Bathing - Playing - Fetching water	- Generic (average) - Washing clothes (riverbank) - Washing utensils - Fishing							- Population dispersion - WASH infrastructure. - Additional public infrastructure	- Gender norms - Payment system		7
**INCENTIVES CENTRED INTERVENTIONS**														
Fink and Rockers 2017 [81]	- Post measures - Equivalent control - Random assignation	- Socioeconomic survey - Medical records					- Treatment seeking		- Wealth					8
Muhumuza et al., 2014 [82]	- Pre-post - Equivalent control - Random assignation	- KAP survey					- Treatment uptake		n.r.	n.r.	n.r.	n.r.	n.r.	8

a. Significant beneficial change: p-value < 0.1

b. No beneficial change: p-value > 0.1

c. Unspecified water-contact activity. Covers any form of interaction with water.

d. Proxy measure for safe water use

e. Proxy measure for open defaecation

One SEI reporting positive outcomes was the ‘Enhanced Development Governance’ intervention in Tanzania [66], which strengthened village-level social services committees (SSCs) so they could implement NTD-WASH initiatives. Over a year, the project expanded SSC’s membership, provided start-up funds, training, and technical support. SSCs organised monthly clean-ups, sensitisation activities, established community sanitation standards, and built wells and latrines. A second effective SEI was a one-year CLTS intervention in Cote d’Ivoire, relying on participatory exercises (e.g., mapping of defaecation areas) to raise awareness on transmission and sanitation as well as trigger emotional responses (e.g., disgust) [64]. Participatory planning sessions followed, where residents settled on strategies to become open-defaecation free (ODF). Technical and material assistance for latrine building was provided. Both projects found that, compared to control groups, beneficiaries reported increased latrine use. Observed measures confirmed that open defaecation decreased, with the CLTS project reporting that four of five intervention villages became ODF.

Transmission and exposure behaviour were targeted by a project in Ghana, where in-depth training was provided to community volunteers, who visited families to provide health education and mobilise residents [63]. Community meetings ensued to outline solutions and action plans for safe water, latrine building and environmental management, subsequently led by local committees. Material and technical support was provided upon demand. The two other study arms consisted of limited education activities (videos, printed materials, and lectures) provided during local associations’ meetings and no education provision. Over 2 years, the intervention arm built more wells, public and private latrines than the comparison groups.

An SEI in Mozambique targeted transmission behaviour, alongside two other risk practices. Volunteers organised ‘community-dialogue’ sessions, where villagers learned about the disease, its health and socioeconomic impacts, and treatment; developed locally applicable solutions; and outlined implementation plans [74]. One solution included latrine building but the project did not provide material support. Additional proposals involved sensitising on and committing to cease bathing or swimming in freshwater, treating water, and seeking treatment. Over a year, exposure-related initiatives attained mixed results, with adults reporting a significant decrease in swimming activities but no changes for bathing; self-reported latrine use did not improve; whilst self-reported MDA coverage improved among children but remained low (<20%). Parents’ treatment attitudes worsened.

The PHAST framework was used in Tanzania to modify exposure behaviour [67,70]. Two successive projects ran participatory workshops with trained community facilitators, where residents were introduced to schistosomiasis; discussed transmission and risks practices; and outlined potential solutions, action plans, goals, and monitoring. The earlier 3-year PHAST project [67] found a significant decrease in adults’ bathing and fetching water (self-reported). Observations of water contact frequency, however, showed unfavourable changes among adults and females (all ages). Overall duration of water contact decreased for all groups. The second project [70] additionally provided pumped wells and described positive changes in adults’ self-reported rates of contact with infected and dirty water, bathing at home, and using safe water sources after two years. The first study also examined the effects of PHAST on treatment seeking, reporting that self-medication with praziquantel improved among women.

A community co-designed project following a ‘human-centred approach’ in Tanzania targeted all three risk practices. This SEI conducted workshops with villagers and teachers to develop teaching materials and school-based activities, identify safe play activities, organise public informative events, as well as design and install urinals and laundry-washing platforms. Materials presented schistosomiasis as a ‘blood fluke’ to distinguish it from regular worms and used striking images to trigger emotional responses. Compared to a control group, retrospective surveys indicated that targeted children (except boys from one island) washed and bathed at surface water less. No outcome measures were reported pertaining open urination. Beneficiary schoolchildren who initially rejected treatment were reportedly more likely to take praziquantel post-intervention [71]. Overall, however, MDA coverage for districts without BC activities was similar to those with [72].

SEIs highlighted community factors as potential barriers for BC. Inadequate social cohesion or governance structures resulted in deficient project implementation, resource access or management, and rule enactment [63,64,66], whilst communities’ limited material and non-material resources (e.g., technical knowledge) conditioned the feasibility or effectiveness of local proposals and their long-term sustainability [64,66,74]. Limited availability of safe water and sanitation infrastructure, including complementary public infrastructure (e.g., latrines close to farmland) was noted to restrain community efforts [63,64,67,70–72,74]. Beneficiaries’ livelihoods, age, sex and gender-based norms were also highlighted since they shape domestic and economic practices and public space use [63,64,67,70,74]. Additionally, SEIs stressed that community-wide change involve long-term processes [64,72,74].

### Effectiveness of physical environmental interventions

All five PEIs reviewed attempted to control exposure behaviour, one also targeting transmission practices. All reported mixed results.

Two PEIs examined how community piped water provision altered water contact practices. One provided five standpipes and a free-of-charge shower unit in Kenya [79] and the other, in Cameroon, installed seven community standpipes [80]. Both projects demanded payment for use. Observed measures in Kenya showed significant reductions in the overall duration and number of people using river water post-intervention. Individual averages, however, indicated non-significant reductions in the frequency and duration of river water contact per person. Frequency of contact decreased for bathing, fetching water, and playing but not for washing clothes or utensils and fishing. In Cameroon, the rate of pregnant women exclusively using stream water decreased. Most (76.1%), however, still used it occasionally for domestic purposes.

Two PEIs likewise targeted exposure BC through different infrastructure developments. In Ghana, a water recreation area was built to encourage safe game activities among children [78]. Post-implementation observations reported low but significant rates of contact with the river (range: 10%-20%). No baseline measures were attained to assess change. In Kenya, borehole wells were provided to villages without access to piped water [76]. Self-reported measures indicated that households reduced high-risk exposure to natural water sources for drinking, cooking, bathing, and washing dishes. Most, however, continued washing clothes in such areas (>70%). Marsh/pond water continued being used as secondary water sources for bathing, washing clothes and playing for over 40% of adults and children.

The PEI focussing on exposure alongside transmission, in Kenya, provided schools with latrines, handwashing and drinking water containers, and water treatment product [77]. One parent and teacher per school received health education and technical training. The project only reported proxy outcome measures, observing no significant differences between intervention and control schools concerning water sources used during the dry season. Beneficiary schools, however, were more likely to provide treated water for drinking and handwashing and marginally improved the average number of latrines per student.

PEIs mainly highlighted technical factors as barriers, including number of facilities built, population dispersion, costs of use or maintenance, and availability of complementary infrastructure (washing or shower stations) [76,79,80]. Environmental factors, like the quality of underground water and proximity to unsafe surface water sources were also mentioned [76,77].

### Effectiveness of incentives-centred interventions

ICIs were used to promote treatment seeking or uptake. A conditional cash-transfer experiment in Zambia offered parents payment if they took their children to a health centre for test-and-treat services within a week [81]. Four comparison groups received varying amounts of money, from 0USD to 3.06USD. Results showed that a 1USD payment increment improved uptake by around 10%. However, wealth affected outcomes. Even with the highest incentive, less than 35% of parents in the wealthier two quintiles followed the advice. A randomised controlled trial in Uganda, in turn, trialled food provision as an incentive for participation and to prevent side effects [82]. Results showed that higher therapeutic coverage was achieved among schools providing a snack pre-treatment (93.9% vs 78.7%).

### BC effects on schistosomiasis epidemiology

The evidence on epidemiological impacts of BC is limited. To assert them, aside from pre-post intervention epidemiological data, studies needed to demonstrate changes in prevalence or intensity did not emanate from treatment activities but from BC, either by not providing praziquantel tablets or using suitable controls. Of 31 projects reviewed, twelve did not gather epidemiological data [47,48,50,54,58,60,62,67,70,74,79,81], one reported no significant BC [45], and eight could not isolate BC’s effects [46,49,52,55–57,64,78] (See Table 5). Only ten projects yielded some evidence; three reporting no favourable epidemiological changes despite some improvements in water contact behaviour (the ‘human-centred’ SEI in Tanzania [72] and borehole provision PEI in Kenya [76]) and in access to treated water and latrines (PEI providing latrine, equipment, and water treatment to Kenyan schools) [77].

**Table 5:** Summary of evidence on reported epidemiological outcomes after BC interventions. To access full results, see S2 Table.

REFERENCE	INFORMATION SOURCE	EPIDEMIOLOGICAL OUTCOMES		DATA CONSIDERATIONS	
		Significant beneficial change ^a^	No significant change ^b^	Praziquantel provided with BC intervention?	Control for pharmacological impacts? ^c^
**HEALTH EDUCATION INTERVENTIONS**					
Chaula & Tarimo, 2014 [45]	Parasitological survey	- Prevalence - Intensity		Yes	No ^c^
Cline & Hewlett, 1996 [46]	Parasitological survey	- Prevalence - Intensity		Yes	No ^d^
Favre et al., 2021 [49]	Parasitological survey		- Prevalence - Intensity	Yes	No ^e^
Hong et al., 2011 [52]	Parasitological survey	- Prevalence.		Yes	No ^f^
Guang-Han et al., 2005 [51]	Parasitological survey	- Reinfection rate (women and children)	- Reinfection rate (men)	Yes	Yes
Jia-Gang et al., 2005 [53]	Parasitological survey	- Prevalence.		Yes	Yes
N’Diaye et al., 2016 [55]	Parasitological survey	- Prevalence.		Yes	No ^f^
Nagi et al. 2005 [56]	Parasitological survey	- Prevalence - Intensity		Yes	No ^f^
Oyeyemi et al. 2018 [57]	Parasitological survey	- Prevalence - Intensity		Yes	No^f^
Wolmarans & de Knock, 2009 [55]	Parasitological survey	- Prevalence - Intensity		Yes	Yes
**SOCIAL ENVIRONMENTAL INTERVENTIONS**					
Nsowah-Nuamah et al. 2001 [63]	Parasitological survey	- Prevalence	- Intensity	Yes	Yes
Hurlimann et al., 2018 [64]	Parasitological survey	- Prevalence		Yes	No^g^
Madon et al., 2018 [66]	Self-assessment questionnaire	- Prevalence		No	N.A.
Knopp et al., 2019 [72]	Parasitological survey		- Prevalence - Intensity	Yes	Yes
**PHYISICAL ENVIRONMENTAL INTERVENTIONS**					
El Kholy et al, 1989 [76]	Parasitological survey		- Incidence	Yes	Yes
Freeman et al., 2013 [77]	Parasitological survey		- Prevalence - Intensity	Yes	Yes
Kosinski, et al., 2016 [78]	Parasitological survey	- Prevalence		Yes	No ^f^
Wepnje et al., 2019 [80]	Parasitological survey	- Prevalence - Intensity		No	N.A.
**INCENTIVES CENTRED INTERVENTIONS**					
Muhumuza et al., 2014 [82]	Parasitological survey	- Prevalence - Intensity		Yes	Yes

a. Significant beneficial change: p-value < 0.1

b. No significant change: p-value > 0.1

c. No baseline measures

d. No intervention vs control comparisons

e. No evidence of significant beneficial BC

f. Single group design

g. Intervention and control groups were not comparable for schistosomiasis prevalence at baseline. Prevalence at intervention group <1% at baseline.

Two HEIs reporting improvements in water contact behaviour indicated reductions in re-infection rates. The intervention in Jiangxi, China [51], comprising lectures, exhibitions, videos, and information boards, reported a significant decrease in re-infection rates during a decade among SAC and women, following their reduced exposure to surface water. Another project, in South Africa, compared three arms: health education with puppets alongside annual MDAs, MDAs only, and no activities [61]. Results showed that prevalence and intensity rates decreased post-intervention and remained low in the intervention group over three years, decreased but mildly bounced back in the MDA-only group, and remained unaltered in the no-activity group.

Additionally, the ‘passive-chemotherapy’ HEI in China [53], reported that over 90% of positive cases self-referred for treatment. A year later, no significant differences in prevalence were thus found with the control group (blanket MDA).

Among SEIs, the Enhanced Development Governance project in Tanzania, which expanded SSCs’ membership and provided technical and financial support [66], reported significant reductions in *S*. *mansoni* and *S*. *haematobium* prevalence among schoolchildren, compared to a control group, following improvements for open defaecation. Parasitological estimates, however, were attained via self-administered questionnaires, thus requiring further validation [84]. A second SEI, in Ghana, contrasted outcomes from community sensitisation and mobilisation work against two comparison villages: MDA-only and MDA with occasional lecture-based health education. Results showed a lower likelihood of infection for the intervention arm, after improvements in wells and latrines availability [63].

Among PEIs, the Cameroon-based project providing community-piped water found significant reductions in disease prevalence and infection intensity among pregnant women over three years, after improvements in safe water use. Prevalence rates, however, remained high (>20%) [80].

The food-based ICI in Uganda reported that the provision of a snack prior school-based MDAs achieved significantly larger treatment coverage than non-incentivised schools, yielding analogous significant reductions in disease prevalence and intensity [82].

## Discussion

### Lessons from BC approaches to control of risk behaviours for schistosomiasis prevention

The WHO considers the development of effective BC interventions critical to achieve the 2030 NTDs roadmap’s goals [11]. For schistosomiasis, changing people’s exposure, transmission, and treatment seeking or uptake practices is expected to accelerate and sustain the reduction in infection prevalence, attained mostly through preventive chemotherapy [14,15]. To assess progress on the impact of BC interventions, this review conducted a comprehensive systematic literature search, assessing 31 projects implemented in LMIC in the last three decades.

The primary objective of this review was to establish the BC strategies being implemented to control schistosomiasis transmission and their effectiveness in altering risk human behaviour. Four general intervention models were identified health education, relying on information-provision to motivate BC; social-environmental, trying to re-shape (in)formal social structures to enable and prompt BC; physical-environmental, using infrastructure and equipment investments to guide behaviour; and incentives-centred interventions, relying on conditional offers of material support. HEI was the most common approach used (n = 17). This trend likely reflects past programmatic efforts to overcome prevalent knowledge gaps among vulnerable groups and motivating support for public health campaigns, chiefly MDAs [34,85].

Despite the evidence gathered, a definitive answer concerning effectiveness remains elusive. One consideration is that the varied indicators used to operationalise risk behaviours render comparisons difficult, with some projects measuring changes in overall water-contact or open defaecation and others using disaggregated (e.g., bathing, washing, fishing) or distinct observations (e.g., urinating “in freshwater”). In addition, substantive reliance on self-reported measures, with twelve projects relying exclusively on them, raised concerns about potential bias; whilst the limited use of theoretical frameworks that explain how and why proposed interventions were expected to prompt BC made it unclear what the specific hypotheses were being tested. Observed results (Table 4) indicate that none of the intervention models identified can be singled out as the most effective in encouraging the adoption of schistosomiasis prevention measures. Promising trends were observed concerning HEIs and ICIs for treatment uptake. However, careful assessment of the advantages and limitations of each approach is needed before programmatic integration, as we discuss next.

An 2015 review of HEIs from Sub-Saharan Africa, including three BC interventions, concluded that whilst education provision improved knowledge and awareness, behavioural impacts remained uncertain [23]. This study, despite reviewing a higher number of projects, agrees with that observation. HEI projects mostly reported mixed results, apart from those pertaining to treatment uptake behaviour. The factors they identified as affecting their impact help to understand those trends. HEIs mostly attempt to motivate individuals by providing information that enhance their perceptions of susceptibility to infection, associated (non)material costs, their severity, and the benefits of prevention. Whilst knowledge-based approaches sometimes result in BC [86,87], it is seldom enough since individuals often need additional resources to redress the conditions that make certain risk behaviours common (e.g., costs associated with changing to a healthier diet) [25,85]. This was reflected in our review. HEIs often identified structural barriers, including traditional socio-cultural practices [45,51,58] and livelihoods [45,51,52,55,58,60], which affect the local use of space and so peoples’ likelihood of exposure and transmission, as well as material conditions, like availability of WASH infrastructure [45,47–50,58,60,62] and the natural environment (e.g., flooding) [51,58,60,62]. Social considerations likewise matter, since risk practices are often embedded in social relations and require individuals to have sufficient agency to contravene social conventions [54,88]. HEIs working exclusively with segmented target audiences (e.g., schoolchildren), may leave some unable to alter the demands of uneducated authoritative figures (e.g., parents’ views on water-based domestic tasks).

Reports of favourable BC by HEIs, correspondingly, can be understood in relation to the absence of aforementioned barriers. Positive changes concerning transmission happened when beneficiaries were recommended to stop urinating in streams or ponds [48,58] or defaecating in agricultural fields [60], rather than stopping open defaecation altogether; all recommendations that do not demand altering economic activities, challenging social traditions, or using new infrastructure or equipment [28,89]. Positive BC outcomes for treatment uptake or seeking behaviour could likewise be associated with treatment activities characterised by free or low-cost drug provision, limited travelling demands (home- or school-based distribution), no demands for equipment (provided by control programmes), and limited challenges to socio-cultural structures [46,47,49,51,53,56,60]. It is thus recommended that future HEIs carefully map out potential barriers across all levels of analysis (individual to societal) to assess what additional components are needed to enable actors to effectively use their new knowledge.

Notwithstanding those considerations, the BC literature considers that the delivery of enticing, precise, and well-framed messages is key to prompt individuals to initiate action [28,90]. Certain pedagogical techniques appeared useful in our review: (i) (audio)visual materials, given their capacity to summarise complex information [47,48,51,71,74] and generate emotional reactions (e.g., frightening images) [71]; (ii) participatory sessions, which help beneficiaries to assess problems and outline solutions themselves, thus enhancing their confidence to take action [67,70,71,74]; (iii) context-based tailored messages, which ensure recommendations are applicable to local realities and relatable to beneficiaries’ concerns [48,51,71,74]; and (iv) enjoyable delivery platforms, like songs or games, which not only enhance their learning experience but can also attach positive emotions to safe practices (e.g., enjoyment) [47,48,71]. Mobilising trusted local figures, like school and religious teachers, has been established to add credibility to health messages [42,51,56]. Future interventions should consider making combined use of these strategies for their educational components.

SEIs, correspondingly, have the potential to tackle some challenges missed by information-based activities. That approach commonly request residents to assess local practices, outline solutions, develop action plans, and set goals [65,66,68,75]. These measures are helpful to overcome the attitude-behaviour gap hampering BC, giving individuals a clear plan on how to manage existing resources to implement recommendations and sidestep or overcome barriers [28]. In addition, by promoting community (participatory) events, mobilising leaders and associations to lead, and sponsoring new norms or standards, SEIs can foster an enabling social environment that supports rather than rejects change [43,74]. Then, social networks, social influence and pressure mechanisms, as well as governance structures synchronised with health recommendations can generate feedback mechanisms that positively reinforce BC, so that safe practices gradually become perceived as the new ‘normal’ or ‘correct’ behaviour. Widespread community mobilisation and cooperation, moreover, can help assemble local resources to support investments that cannot be otherwise made by single individuals (e.g., public infrastructure), whilst enhancing the involvement of otherwise hard-to-reach or marginalised populations, possibly leading to improved health equity [65]. Additionally, by asking residents to propose solutions and lead their implementation as well as providing training and technical support, SEIs can enhance people’s perception of their capacity to overcome barriers and achieve goals, a crucial condition for sustained BC [28].

Despite their theoretical potential, our review showed uneven outcomes from SEIs, with three of seven reporting mixed results for exposure and treatment uptake [69,71,72,74]. An important distinction between these and the SEIs reporting positive outcomes concerned the provision of technical, financial, or infrastructure support to community-led initiatives, as compared to relying heavily or exclusively on locally-available resources [63,64,66,70]. The significance of this was observed in the SEI in Mozambique, which found that lack of technical expertise and material poverty affected, respectively, the range of solutions residents could outline to deal with transmission and their ability to build the latrines initially planned [74]. Proponents of SEI, in this regard, should be mindful that NTDs are diseases of ‘poverty’, endemic to places characterised by their geographical isolation, limited access to public services, and material deprivation [11]. Debates on community-based development have long established that there is a limit to the amount of economic, social, and technical resources that impoverished communities can mobilise. Stable and formal relationships with (public) development agencies (vertical integration or ‘linking’ social capital) is required to ensure that local initiatives can take-off, be scaled-up, and sustained over time [91,92].

Programmes aiming to implement SEIs are recommended to carefully assess local socio-economic and governance conditions before deployment. This and previous reviews of SEI and community-based health promotion interventions suggest effectiveness is context-sensitive [66,74,93,94]. Weak social cohesion can affect local capacity for collective-action; inadequate governance may generate accountability problems; local forms of inequality can be reproduced in newly established organisations, whilst limited engagement with (or trust in) public health programmes can leave communities unable to formally support mechanisms to ensure sustainability.

A major barrier shared by HEIs and SEIs concerned the need for significant investments in infrastructure or equipment to support BC, chiefly for exposure and transmission behaviours [45,47–49,58,67,70]. Similarly to previous studies, two main considerations were noted. One is that such investments should implement a users’ perspective to ensure adoption [43,95]. Practicality of designs, location of new facilities, demand levels, affordability, and local capacity for maintenance are all issues that require consultation with beneficiaries. For instance, if personal protective equipment hampers agricultural work, wells provide hard water that makes washing difficult, or if boreholes cannot provide the volumes of water required for bathing, associated preventive measures will not be adopted [60,76,79]. A second consideration pertains the need for comprehensive material investments. Local adoption of infrastructure or equipment may not fully support schistosomiasis control unless they are understood as part of a wider system of interconnected processes and complementary practices [19,36,96]. For instance, due to the volume of water needed for bathing and washing clothes, additional ad-hoc facilities may need to be built alongside boreholes and wells; WASH facilities in public areas, including areas of work, could be as important as in private homes, since exposure and transmission happen there; and ensuring that adequate maintenance, hygiene, and waste disposal mechanisms are in place could be as important as latrine use since transmission may still occur if infrastructure is poorly installed or maintained. Whilst technical considerations are likewise relevant to treatment uptake behaviour, the NTD’s sector decades of experience implementing PC interventions have allowed for the development of many cost-efficient solutions (e.g., dose-poles) [13,97], with further refinements in drugs and diagnostics in the pipeline (e.g. paediatric praziquantel formula) [98].

Finally, the evidence concerning ICIs remains insufficient. Available data from two projects in this study, however, coincided with trends observed by a recent review of conditional cash-transfers applied to NTDs control [99]. One is that conditional transfers appear to attain greater reach among lower socioeconomic groups but, whilst that may help to reduce health inequalities, the epidemiological contributions of those skewed effects remain unclear [81]. Additionally, to date, there is insufficient evidence to confirm the cost-effectiveness and long-term sustainability of this approach. A follow-up study for the snack-provision trial in Uganda, for instance, showed that gains in therapeutic coverage were quickly lost once food support was withdrawn [100]. Further research is needed.

**Fig 5:**
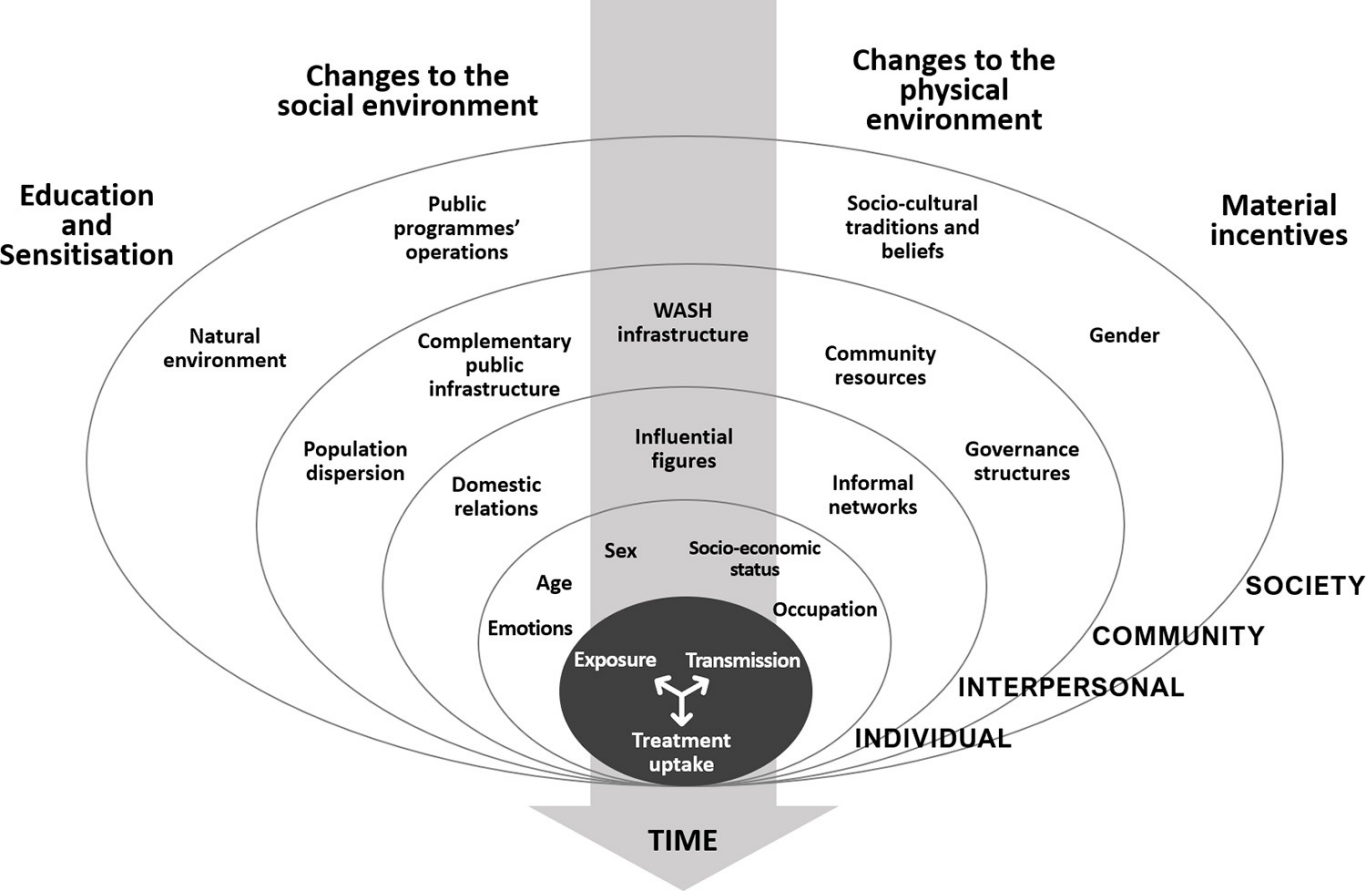
Factors affecting the effectiveness of existing BC strategies for schistosomiasis control.

In summary, evidence on the effectiveness of intervention models indicate that there is substantive need for integrated knowledge-based, social and physical environmental approaches to BC for schistosomiasis elimination. It has been proposed by various frameworks that BC is likely to occur when people have the capability (physical and psychological), the opportunity (enabling social and physical contexts), and motivation to act (through cost-benefits evaluations and emotional impulses) [28,90,95]. Integrated approaches could ensure such conditions. However, the specific combination of techniques and components to be used require preliminary assessment and planning, both to understand their contribution to the specific type of behaviour to be targeted and how local socio-cultural and ecological conditions may affect BC. In all cases, it is apparent that no quick solutions are available, BC efforts require long-term investments (Fig 5).

### Epidemiological impacts of BC interventions

Evidence concerning the epidemiological impacts of BC, our secondary objective, remains limited (six studies). Available information somewhat indicates that BC is more likely to boost, in the short-term, and sustain, in the long-term, the epidemiological gains attained through pharmaceutical interventions rather than generate those gains independently. Only two projects suggested the latter but with significant caveats. A SEI in Côte d’Ivoire reported a decrease in prevalence following a reduction in open defaecation practices but epidemiological estimates were attained via a self-administered questionnaire [66], whilst a PEI in Cameroon observed a reduction in prevalence and intensity after a reduction in stream water use but prevalence rates three years post-intervention remained high (>20%) [80]. The rest complemented the effects of PC activities. In Uganda, boosted treatment uptake through snack provision lead greater reductions in infection prevalence and intensity compared to MDA alone; education for self-referral in China preserved low prevalence rates after the suspension of blanket MDAs (<10%) the two remaining studies improving water contact behaviour resulted in low re-infection rates among target groups over ten (China) and three years (South Africa) [51,61]. These results thus support ongoing calls for developing programmes integrating PC and BC components to reduce infection prevalence and, eventually, eliminate schistosomiasis [13–15].

### Limitations

This review faced various limitations. First, there was geographical bias in the selected interventions, three-quarters of which were based in Africa (24 of 31) and a similar proportion based in rural areas exclusively (n = 25). These trends were also observed in other reviews on schistosomiasis control [30,32,36,37], indicating they mostly mirror the concentration of control efforts in highly endemic areas [11].

Next, methodological limitations were noted in part of the reviewed interventions. These included the absence of behavioural indicators (5 of 31), exclusive reliance on self-reported measures (n = 14), and lack of control groups (n = 10). Whilst we are aware that some articles could have been discarded based on quality assessment measures, setting a specific inclusion threshold was not considered suitable given the variety of intervention and evaluation designs. We opted instead for an inclusive strategy given the scoping and narrative nature of this review [41]. Most studies (n = 16) provided sound evidence (quality assessment scores ≥7).

A third consideration pertains the classification of approaches. Most projects lacked theoretical frameworks (24 of 31) or explanations of how their components were expected to re-configure beneficiaries’ behaviour. The team thus systematically examined the project’s BC techniques to outline four main intervention models, drawing on an existing classification [43]. Grouped projects were then assumed to possess a broadly similar rationale. Quality measures were adopted to ensure the reliability of this classification, including working in pairs during coding and conducting multiple review rounds. We are aware, however, that other typologies could be used.

## Conclusion

Our review highlights the need for investments in integrated BC interventions. Combining appropriate pedagogical techniques for HEIs with community-based planning and user-defined infrastructure components may both motivate and enable beneficiaries to adopt recommended practices as well as addressing or circumventing structural barriers, fostering an enabling social and physical environment, to sustain the new behaviours. This appears central to encourage safe water contact practices, reduce transmission behaviours and, promote treatment seeking or uptake. Whilst further research is needed to ascertain the costs of integrated approaches, chiefly when considering that fostering BC may require long-term support, indicative evidence that BC can help sustain low infection rates post-MDAs suggest that such investments may be cost-effective as part of programmes’ elimination plans.

Methodological and data gaps noted in the literature, outline key considerations for further research. Projects/programmes need to be clear on the type of behaviour targeted, the rationale/theory behind the intervention design, the specific contributions of different intervention components or BC techniques, and the pathways through which BC is expected to generate epidemiological impacts. Accompanying evaluation studies would benefit from using control groups and behavioural indicators validated through observed behaviours, discussions or interviews, as well as reliable diagnostic tests. Evaluations should also be mindful of the longevity of BC processes. Projects with these components will greatly contribute to the body of evidence for BC approaches for schistosomiasis elimination.

## Supporting information

S1 PRISMA Checklist PRISMA 2020 Checklist. (DOCX)

S1 Table Quality assessment criteria. (DOCX)

S2 Table Complete results from selected behaviour change interventions. (DOCX)
