# Supplementary material for: Behaviour change interventions for the control and elimination of schistosomiasis: A systematic review of evidence from low- and middle-income countries
Source: PLoS Negl Trop Dis. 2023 May 10;17(5):e0011315. doi: 10.1371/journal.pntd.0011315 (PMC10202306; doi:10.1371/journal.pntd.0011315)
Supplement: S2 Table — (DOCX) [file pntd.0011315.s003.docx]

S2 Table. Complete results from selected behaviour change interventions

| **Reference** | **Length of evaluation** | **Design** | **Treatment for comparison group** | **Methods** | **Methods and sample size** | **BCC-related**  **outcome measures** | **Outcomes** | **Conditioning Factors** |
| --- | --- | --- | --- | --- | --- | --- | --- | --- |
| Chaula & Tarimo, 2014 [1] | - 2011 – 2013^(a)^ | - Post measures - Non-equivalent groups - Random assignation | - Control from same school as intervention. - No health education - No treatment | - KAP survey - Parasitological survey | a. Intervention:   - n-schools= 5 - n-schoolchildren= 260.   b. Comparison:   - n-schools= 5 - n- schoolchildren = 228. | Exposure | 1. Perc. of children using safe water sources for domestic use (self-reported)  - Advocacy group: 84.2% - Non-advocacy group: 90.4% - X^2^= 4.047, p=0.055  1. Perc. of children who had contact with unsafe water bodies (self-reported).  - Advocacy: 100% - Non-advocacy group: 100% | - Occupation: rice farming - Socio-cultural: traditional roles and lifestyles. - Infrastructure: lack of safe water sources. - Time: Long-term process. - Emotions: Fear of side effects. |
|  |  |  |  |  |  | Treatment uptake | 1. Perc. of schoolchildren that received Praziquantel during MDA:  - Year 2011: 39.5% - Year 2012: 43.6% |  |
|  |  |  |  |  |  | Epidemiological: | 1. Prop. of pupils with positive diagnosis.  - Advocacy group: 3.1% - Non-advocacy group: 28.5% - X^2^=61.77, p<0.001  1. Prop. of pupils with heavy intensity infections ^a^.  - Advocacy group: 3.1% - Non-advocacy group: 28.5% - X2=61.77, p<0.001 |  |
| Cline & Hewlett, 1996 [2] | - 1991-1993 | - Pre-post measures - Non-equivalent groups - Non-random assignation | - No health education - Treatment provided | - Treatment reports - Parasitological survey - KAP survey | n-villages=4  n-individuals= 651 (BL and FU, matched) | Exposure | N.R. | - Community engagement: support of community groups for educational activities. - Population dispersion - Public programmes engagement: support of local health centres - Health staff training |
|  |  |  |  |  |  | Transmission | N.R. |  |
|  |  |  |  |  |  | Treatment seeking | No. of outpatient consultations for schistosomiasis in local health centres: 167 (BL), 172 (FU1), 1261 (FU2)^(b)^ |  |
|  |  |  |  |  |  |  | No. of positive cases for schistosomiasis diagnosed in local health centres: 167 (BL), 174 (FU1), 1206 (FU2)^(b)^ |  |
|  |  |  |  |  |  | Epidemiological | Perc. of residents positive for *S. haematobium* in assessment villages: 21% (BL), 7% (FU) ^(b)^ |  |
|  |  |  |  |  |  |  | Perc of residents with heavy intensity infections for *S. haematobium*: 23% (BL), 8% (FU) ^(b)^ |  |
| Ejike et al. 2017 [3] | - Ap – Aug 2014 | - Pre-post measures - Equivalent groups - Random assignation | - Control from same school as intervention. - No health education. | KAP survey | a. Intervention:   - n-school = 1 - n-schoolchildren = 50 (BL), 50 (FU).   b. Control:   - n-school = 1 - n-schoolchildren = 50 (BL), 50 (FU). | Exposure | a. Daily frequency of exposure to dam water (self-reported)   - Intervention - Twice or thrice: 42.0% (baseline), 82% (FU). - Intervention - More than thrice: 58.0% (baseline), 18% (FU). - Control -Twice or thrice 36.0% (baseline), 60% (FU) - Control - More than thrice: 64.0% (baseline), 40% (FU) - Pre-test X^2^ : p=0.278 - Post-test X^2^: p=0.048.   b. Daily frequency of playing activities in dam water (self-reported)   - Intervention - Always: 84.0% (baseline), 16.0% (FU) - Control - Always: 88.0% (baseline), 8.0% (FU) - Intervention – Not always: 6% (baseline), 80% (FU) - Control – Not always: 6% (baseline), 92% (FU) - Intervention - Never: 10% (baseline), 4% Never (FU) - Control - Never: 6% (baseline), 0% (FU) - Pre-test X^2^, p=0.761 - Post-test X^2^, p=0.153 | - Household: Parents order children to do water-related chores. - Infrastructure: Lack of safe water sources. - Networks: children share information regularly. - Study design: Cross-contamination across groups. |
| Ejike et al., 2021 [4] | - Oct 2018-Mar 2019 | - Pre-post measures - Equivalent groups. - Random assignation. | - No health education | - KAP survey | a. Intervention:   - n-school = 3 - n-schoolchildren = 98 (BL), 78 (FU)   b. Control:   - n-school = 3 - n-schoolchildren= 177 (BL), 177 (FU) | Exposure | a. Perc. of schoolchildren wearing rubber boots (self-reported)  - Intervention: 3.1% (baseline), 9.0% (FU)  - Control: 4.5% (baseline), 4.5% (FU)   - Baseline X^2^, p=0.343 - Post-intervention X^2^, p<0.001   b. Perc. of schoolchildren fishing in river (self-reported)  - Intervention: 8.2% (baseline), 9.0% (FU)  - Control: 9.0% (baseline), 1.7% (FU)   - Baseline X^2^, p=0.780 - Post-intervention X^2^, p=0.880   c. Perc. of schoolchildren washing in river (self-reported)   - Intervention: 87.8% (baseline), 66.7% (FU) - Control: 96.6% (baseline), 96.6% (FU) - Baseline X2, p=0.172 - Post-intervention X2, p=0.019   d. Perc. of schoolchildren fetching water from river (self-reported)   - Intervention: 78.6% (baseline), 66.7% (FU) - Control: 90.4% (baseline), 90.4% (FU) - Baseline X2, p=0.008 - Post-intervention X2, p=0.001   e. Perc. of schoolchildren bathing in river (self-reported)   - Intervention: 89.8% (baseline), 64.1% (FU) - Control: 97.7% (baseline), 97.7% (FU) - Baseline X2, p=0.709 - Post-intervention X2, p=0.085 | - Domestic: Supports of parents / caregivers is needed to implement recommendations. - Infrastructure: Lack of safe water sources. - Infrastructure: Lack of public sanitation infrastructure. |
|  |  |  |  |  |  | Transmission | a. Perc. of schoolchildren practicing open urination (self-reported)   - Intervention: 80.6% (baseline), 60.3% (FU) - Control: 63.3% (baseline), 63.3% (FU) - Baseline X2, p=0.024 - Post-intervention X2, p=0.001   b. Perc. of schoolchildren practicing open defecation (self-reported)   - Intervention: 11.2% (baseline), 9.0% (FU) - Control: 33.3% (baseline), 33.3% (FU) - Baseline X2, p=0.017 - Post-intervention X2, p=0.110 |  |
|  |  |  |  |  |  | Treatment seeking / uptake | a. Perc. of schoolchildren that previously rejected treatment that registered to receive it (observed)  - Intervention: 0% (baseline), 65.3% (FU) |  |
| El Kholy et al., 1989 [5] | - 1984-1987 | - Post measures - Non-equivalent control. - Non-random assignation | - No boreholes, villages had piped water. | - KAP surveys - Parasitological surveys. | a. Intervention   - n-villages = 3 - n-households (KAP)= 47 - n-children (Epidemiological)=46   b. Control:   - n-villages = 2 - n-households (KAP)= 67   n-children (Epidemiological)=112 | Exposure | a. Perc. of households using high-risk water sources for drinking/cooking (self-reported): 18% (Pre), 2%(Post)^(b),^ p<0.02  b. Perc. of households using high-risk water sources for washing dishes (self-reported): 20%(Pre), 6% (Post)^(b)^, p<0.05  c. Perc. of households using high-risk water sources for bathing (self-reported): 43%(Pre), 72%(Post)^(b)^, p<0.05  d. Perc. of households using high-risk water sources for washing clothes (self-reported): 80%(Pre), 72%(Post), p>0.05  e. Perc. of adults using surface water for drinking / cooking (self-reported): ^(b)^  - Intervention: 2%  - Control: 0%  p>0.05  f. Perc. of adults using surface water for bathing (self-reported) ^(b)^:  - Intervention: 53%  - Control: 37%  p>0.05  g. Perc. of adults using surface water for washing clothes (self-reported) ^(b)^  - Intervention: 70%  - Control: 42%  P<0.005  h. Perc. of children (<15) using surface water for drinking / cooking (self-reported) ^(b)^  - Intervention: 2%  - Control: 0%  p>0.05  g. Perc. of children (<15) using surface water for bathing (self-reported) ^(b)^  - Intervention: 43%  - Control: 31%  P<0.02  h. Perc. of children (<15) using surface water for washing clothes (self-reported)^(b)^  - Intervention: 43%  - Control: 24%  p>0.003  i. Perc. of children (<15) using surface water for playing (self-reported) ^(b)^  - Intervention: 38%  - Control: 37%  p>0.05 | - Infrastructure: Limited number of boreholes. - Infrastructure: lack of washing stations or distribution mechanisms. - Rurality: population dispersion. - Costs: financial payments for use / maintenance. - Environment: quality of water available |
|  |  |  |  |  |  | Epidemiological | a. Incidence of infection among schoolchildren ^(b)^  - Intervention: 22% (1984 1985), 28% (1986-1987), p>0.05  - Control: 14% (1984-1985), 5% (1986-1987), p=0.002 |  |
| Favre et al., 2021 [6] | - 2013-2015 | - Pre-post measures - Equivalent groups - Random assignation | - No health education - Targeted treatment | - KAP survey - Parasitological survey | a. Intervention:   - n-school = 4 - n-schoolchildren (KAP)= 153 - n-schoolchildren (parasitological) = 343 (BL), 306 (FU1), 307 (FU2)   b. Control:   - n-school = 4 - n-schoolchildren (KAP)= 123 - n-schoolchildren (parasitological) = 424 (BL), 326 (FU1), 250 (FU2) | Exposure | a. Perc. of schoolchildren that had contact with water bodies   - Intervention: 59.0% (baseline), 56.1% (1-month FU), 60.4 (6-month FU), 59.0% (12-month FU), 55.7% (24-month FU) - Control: 52.0% (baseline), 44.0% (1-month FU), 52.6 (6-month FU), 49.1% (12-month FU), 45.7% (24-month FU). - X2 I-C comparison: p=0.96(BL); p=0.91 (1-month FU); p=0.25 (6-month FU); p=0.58 (12-month FU); p=0.97 (24 month FU) | - Infrastructure: lack of safe water sources. - Rurality: population dispersion / remoteness. - Emotions: Fear of side effects and bitter taste. |
|  |  |  |  |  |  |  | b. Perc. of schoolchildren reporting risk behaviour during water contact (washing animals / vehicles /clothes /utensils, swimming, fishing, collecting sand, bathing).   - Intervention: 87.5% (baseline), 78.9% (1-month FU), 74.0% (6-month FU), 77.2% (12-month FU), 67.5% (24-month FU). - Control: 85.3% (baseline), 73.7% (1-month FU), 71.2% (6-month FU), 71.8% (12-month FU), 72.4% (24-month FU). - X^2^ I-C comparison: p=0.66(BL); p=0.49 (1-month FU); p=0.70 (6-month FU); p=0.37 (12-month FU); p=0.44 (24 month FU) |  |
|  |  |  |  |  |  | Treatment seeking / uptake | c. Perc. of schoolchildren who adhered to stool testing and treatment   - Intervention : 84.1% (baseline), 73.9% (12-month FU), 74.2 (24-month FU) - Control : 81.1% (baseline), 62.3% (12-month FU), 47.8% (24-month FU) - X2 I-C comparison: p = 0.48 (baseline), p = 0.00 (12-month FU), p < 0.001 (24-month FU) |  |
|  |  |  |  |  |  |  | d. Perc. of schoolchildren who adhered to stool testing with a positive S. Mansoni diagnosis   - Intervention 30.3% (CI 95%: 23.5%–37.5%) (baseline), 12.7% (CI 95%: 8.7%–17.6%) (FU1), 6.8% (95% CI: 3.9%–10.7%) (FU2) - Control: 21.8% (CI 95%: 15.1% - 29.4%) (baseline), 4.9% (95% CI: 2.5%–8.2%) (FU1), 2.4% (95% CI: 0.7%–5.5%) |  |
|  |  |  |  |  |  |  |  |  |
|  |  |  |  |  |  | Epidemiological | e. Perc. of schoolchildren positive for schistosomiasis  - Intervention: 30.3 0.3% (CI 95%: 23.5%–37.5%) (BL), 12.7% (CI 95%: 8.7%–17.6%) (FU1), 6.8% (95% CI: 3.9%–10.7%) (FU2).  - Control: 21.8% (CI 95%: 15.1% - 29.4%) (BL), 4.9% (95% CI: 2.5%–8.2%) (FU1), 2.4% (95% CI: 0.7%–5.5%) (FU2).  f. Mean number of eggs per gram of stool among positive cases   - Intervention: 54.2 (95% CI: 35.1–73.3) (baseline), 15.3 (95% CI: 6.0–24.6 epg) (12-month FU), 4.6 epg (95% CI: 1.3–7.9) (24-month FU) - Control : 38,4 (95% CI: 12.9–64.0) (baseline), 7.5 epg (95% CI: −0.7–15.7) (12-month FU), 1.3 epg (95% CI: −0.3 - 3.0) (24-month FU) |  |
| Fink and Rockers 2017 [7] | - July 2011 | - Post measures - Equivalent groups - Random assignation | - Four groups: - No incentive - Payment: 0.41US$ - Payment: 1.43US$ - Payment: 3.06US$. | - Household survey - Medical record review | a. No incentive group:   - n-households = 175   b. Group 0.41 US$:   - n-households = 121   c. Group 1.43 US$:   - n-households = 92   d. Group 3.06 US$   - n-households = 134 | Treatment seeking / uptake | a. Probability of attending check-up: Model 1:   - Any incentive, b= 0.100, p<0.01 - Wealth Q2 / Q1: b= -0.01, p>0.1 - Wealth Q3 / Q1: b= -0.16, p>0.1 - Wealth Q4 / Q1: b= -0.18, p<0.1 - Wealth Q5 / Q1: b= -0.32, p<0.05 - RSq: 0.397   b. Probability of attending check-up: Model 2:   - Incentive (0.41US$ / no incentive), b=0.118, p>0.1 - Incentive (1.43US$ / no incentive), b=0.290, p<0.05 - Incentive (3.06US$ / no incentive), b=0.320, p<0.01 - Wealth Q2 / Q1: b= -0.00 p>0.1 - Wealth Q3 / Q1: b= -0.15, p>0.1 - Wealth Q4 / Q1: b= -0.18, p<0.1 - Wealth Q5 / Q1: b= -0.33, p<0.05 - RSq: 0.408   c. Probability of attending check-up: Model 3:   - Any incentive: b= 0.016, p>0.1 - Wealth Q2 / Q1: b=-0.18, p<0.1 - Wealth Q3 / Q1: b= -0.35, p<0.01 - Wealth Q4 / Q1: b= - 0.31, p<0.01 - Wealth Q5 / Q1: b= -0.33, p<0,05 - Q2 x incentive / Q1 x incentive: b=0.158, p<0.05 - Q3 x incentive / Q1 x incentive: b=0.165, p<0.05 - Q4 x incentive / Q1 x incentive: b=0.104, p>0.1 - Q5 x incentive / Q1 x incentive: b=0.021, p>0.1  1. RSq: 0.426 | - Wealth: Time-costs high for wealthier households but increasing payment meaningful for the worse-off. |
| Freeman et al., 2013 [8] | - May 2007 - Feb 2009 | - Pre-post measures - Equivalent groups - Random assignation | - Targeted treatment - No health education, infrastructure or technical support. | - Direct observation - Parasitological survey | a. Intervention:   - n-schools= 20 - n-schoolchildren = 470 (BL).   b. Control:   - n-schools= 20 - n- schoolchildren = 445 (BL).   c. Intervention & Control ^b^   - n-schoolchildren (FU1) = 975   n-schoolchildren (FU2) =1170 | Exposure | a. Perc. of schools offering water from improved sources   - Intervention: 37% (baseline), 40% (FU2) - Control: 44% (baseline), 58% (FU2) - I-C comparisons at FU2: p=0.63   b. Perc. of schools offering drinking water   - Intervention: 32% (baseline), 85% (FU2) - Control: 44% (baseline), 32% (FU2) - I-C comparisons at FU2: p<0.01   c. Perc. of schools with hand washing water available   - Intervention: 5% (baseline), 85% (FU2) - Control: 22% (baseline), 32% (FU2) - I-C comparisons at FU2: p<0.01   d. Perc. of schools with soap available   - Intervention: 0% (baseline), 25% (FU2) - Control: 0% (baseline), 0% (FU2) - I-C comparisons at FU2: p<0.01 | - Gender norms. - Environmental: Proximity to surface water sources. - Organisational capacity / efficiency: implementation and timing guidance not always followed. |
|  |  |  |  |  |  | Transmission | e. Average pupils per latrine   - Intervention: 89 (baseline), 32 (FU2) - Control: 60 (baseline), 52 (FU2) - I-C comparisons at FU2: p=0.07 |  |
|  |  |  |  |  |  | Epidemiological: | a. Prop. of pupils with positive S Mansoni diagnosis.   - Intervention: 6.1% (baseline), 7.8% (FU1), 9.7% (FU2). - Control: 2.7% (baseline), 4.9% (FU1), 4.8% (FU2) - I-C differences in differences at FU1, p=0.18 - I-C differences in differences at FU2, p=0.69. - I/C odds of positive S. mansoni diagnosis at FU1: OR=1.05, 95%CI (0.44–2.52), p=0.90 - I/C odds of positive S. mansoni diagnosis at FU2: OR=1.52 95%CI (0.39–5.95), p=0.54 - I/C odds of positive S. mansoni diagnosis at either FU1 or FU2: OR=1.29, 95%CI (0.45–3.66), p=0.49.   b. Mean number of eggs per gram of faeces among pupils with positive diagnosis.   - Intervention: 13.6 (baseline), 13.6 (FU1), 31.9 (FU2). - Control: 12.6 (baseline), 8.5 (FU1), 15.2 (FU2) - I-C differences in differences at FU1, p=0.98 - I-C differences in differences at FU2, p=0.62. - I/C rate ratio of mean number of eggs per gram at FU1: RR=1.04, 95%CI (0.37–2.96), p=0.93. - I/C rate ratio of mean number of eggs per gram at FU2: OR=0.47 95%CI (0.13–1.64), p=0.23. - I/C rate ratio of mean number of eggs per gram at either FU1 or FU2: RR=0.63, 95%CI (0.23–1.70), p=0.35 - I/C rate ratio of mean number of eggs per gram at either FU1 or FU2 - GIRLS: RR=1.11, 95%CI (0.26–4.66), p=0.88 |  |
| Garba et al., 2001 [9] | - Feb – Mar 1997 | - Post measures - Non-equivalent groups - Non-random assignation | - No health education | - KAP survey | a. Intervention:   - n-villages = 5 - n-villagers = 323   b. Control:   - n-villages = 1 - n- villagers = 577 | Exposure | 1. Perc. of residents that reported adopting at least one form of prevention against unsafe water contact:   - Intervention: 33%  - Control: 12.2%  - X^2^-test N.R., p<0.01 | - Education: Residents’ limited education. - Social networks: Information sharing through word of mouth. - Infrastructure: Lack of safe WASH infrastructure. |
|  |  |  |  |  |  |  | 1. Perc. of residents that bathe at home:   - Intervention: 21.7%  - Control: 8.3%  - X^2^-Test N.R., p<0.01 |  |
|  |  |  |  |  |  |  | 1. Perc of villagers that bathe in surface water   - Intervention: 78.9%  - Control: 91.3%  - X^2^-Test N.R., p<0.01 |  |
| Guang-Han et al., 2005 [10] | - 1992 - 2003 | - Pre-post measures - Equivalent groups - Random assignation | - No health education - MDA of praziquantel | - KAP survey - Parasitological survey | a. Intervention - KAP:   - n-villages = 4 - n-schoolchildren (6-15) = 89 (BL), 89(FU) - n- women (>15)= 103 (BL), 99 (FU) - n- men (>15)= 89 (BL), 88 (FU)   b. Control-KAP:   - n-villages = 2 - n- schoolchildren (6-15)= 81 (BL), 81 (FU) - n-women (>15)= 101 (BL), 103 (FU) - n-men (>15)= 98 (BL), 112 (FU)   c. Intervention - epidemiological:   - n-villages = 3 - n- schoolchildren (6-15) = 579 (BL), 571 (FU) - n-women (>15)= 674 (BL), 658 (FU) - n-men (>15)= 676 (BL), 687 (FU)   c. Control- epidemiological:   - n-villages = 1 - n- schoolchildren (6-15) = 151 (BL), 142 (FU) - n-women (>15)= 171 (BL), 167 (FU)   n-men (>15)= 183 (BL), 181 (FU) | Exposure | 1. Perc. of beneficiaries conducting water contact activities^(f)^  - Intervention - SAC: 12.4% (baseline), 0.6% (FU) - Intervention - Women: 7.2% (baseline), 5.6% (FU) - Intervention - Men: 25.1% (baseline), 36.9% (FU). - Control: No results explicitly provided for control group (only a graph). | - Occupation: fishing and agriculture (men). - Socio-cultural: gender (women’s patterns of socialisation) and children’s playtime routines. - Soc. influence: Teachers’ prestige - Environment: topography limits technological solutions, manual labour needed. - Emotions: fear of side effects. |
|  |  |  |  |  |  | Treatment seeking / uptake | 1. Perc. of beneficiaries that received treatment.  - Intervention - SAC: 68.5% (baseline), 98.9% (FU), X^2^=30.36, p<0.001 - Control - SAC: 70.4% (baseline), 61.7% (FU), X2=1.35, p=0.245. - Intervention - women: 52.4% (baseline), 92.9% (FU), X^2^=41.33, p<0.001 - Control - women: 60.4% (baseline), 50.5% (FU), X^2^=2.03, p=0.155. - Intervention - men: 75.3% (baseline), 92.1% (FU), X^2^=9.71, p=0.003 - Control men: 81.6% (baseline), 69.6% (FU), X^2^=4.03, p=0.045.  1. Prop. of beneficiaries with positive attitudes towards PC  - SAC – Intervention: 55.1% (baseline), 98.9% (FU), X^2^=48.2, p<0.001 - SAC – Control: 51.9% (baseline), 49.4% (FU), X^2^=0.1, p=0.753. - Women - Intervention: 32% (baseline), 91.9% (FU), X^2^=76.36, p<0.001 - Women – Control: 31.7% (baseline), 28.2% (FU), X^2^=0.3, p=0.582. - Men - Intervention: 67.1% (baseline), 97.5% (FU), X^2^=26.0, p<0.001   Men – Control: 72.6% (baseline), 74.3% (FU), X2=0.07, p=0.791. |  |
|  |  |  |  |  |  | Epidemiological: | 1. Prop. of targeted SAC and adults re-infected with S. japonicum one year after intervention.  - Intervention - SAC: 13.5% (baseline), 2.2% (FU), X^2^=7.75, p=0.005 - Control - SAC: 13.6% (baseline), 18.5% (FU), X^2^=0.73, p=0.392. - Intervention - Women: 19.4% (baseline), 7.1% (FU), X^2^=6.51, p=0.011 - Control - Women: 15.2% (baseline), 21.1% (FU), X^2^=1.14, p=0.285. - Intervention - Men: 28.1% (baseline), 41.4% (FU), X^2^=3.65, p=0.056 - Control - Men: 19.4% (baseline), 25.0% (control), X^2^=0.95, p=0.330. |  |
| Hong et al., 2011 [11] | 2005 - 2008 ^(a)^ | - Pre-post measures - Single group - Non-random assignation. | - N.A. | Parasitological survey | - N-villages = 8 - N-individuals (>5)= 8661 (baseline), 8100 (FU1), 7075 (FU2), 9016 (FU3). | Exposure | N. R. | - Occupation: fishers and sailors. - Migration: Migration from endemic areas. |
|  |  |  |  |  |  | Treatment seeking / uptake | N. R. |  |
|  |  |  |  |  |  | Epidemiological | 1. Percentage of 6-60 population with positive diagnosis for S. japonicum(observed):  - Baseline = 9.0% - FU1=6.5% - FU2=4.6% - FU3=3.2% - X^2^ (baseline – FU3) =293.84, p<0.01. |  |
| Hürlimann et al., 2018 [12] | - July 2011 - Aug 2012. | - Pre-post measures - Non-equivalent groups. - Random assignation. | - Targeted treatment - No planning, health education or technical support. | - KAP survey - Direct observation - Parasitological survey | a. Intervention:   - n-villages = 4 - n-villagers (>5) = 385 (matched BL – FU)   b. Control:   - n-villages = 5 - n- villagers (>5) = 425 (matched BL – FU) | Transmission: | 1. Perc. of HHs that own latrines (observed) **^(f)^**  - Control: 26.3% (baseline), 32.3% (FU) - Intervention: 8.1% (baseline), 95.8% (FU).  1. No. of villages that achieved ODF status (observed)  - ODF at end of project - Control: 0. - ODF at end of project - Intervention: 4/5.  1. Perc. of villagers conducting open defecation (self-reported)  - Intervention: 95.8% (baseline), 44.6% (FU), OR=0.04, 95%CI (0.01–0.12), p<0.01. - Control: 92.3% (baseline), 81.3% (FU), OR=0.31, 95%CI (0.13–0.67), p<0.01 - I/C odds at FU: OR=0.19, 95%CI (0.12-0.30), p<0.001.   e. Perc. of villagers using toilets (self-reported)   - Intervention: 15.48% (baseline), 94.64% (FU), OR=134.00, 95%CI(23.64–5331.72), p<0.001 - Control: 46.7% (baseline), 40.1% (FU), OR=0.43, 95%CI (0.17–0.98), p=0.04 - I/C odds at FU: OR=26.38, 95%CI (12.66-54.96), p<0.001.   f. Perc. of villagers exclusively using toilets (self-reported)   - Intervention: 12.5 to 79.2%; OR=17.00, 95%CI (8.00–43.20), p<0.001 - Control: 26.92% to 29.67%, OR=1.28, 95%CI (0.66–2.51), p>0.05 - I/C odds at FU: OR=9.01, 95%CI (5.52-14.7), p<0.001   g. Perc. of villagers washing hands after defection.   - Intervention: 14.88% (baseline), 41.07% (FU); OR=3.93, 95%CI (2.20–7.47), p< 0.001 - Control: 29.67% (baseline), 25.82% (FU), OR=0.83, 95%CI (0.50–1.34), p>0.05 - I/C odds at FU: OR=2.0, 95%CI (0.25-3.15), p=0.003   h. Perc. of children defecating in latrines.   - Intervention: 8.92% (baseline), 11.97% (FU); OR: 58.00 95%CI (15.70–484.61), p< 0.001. - Control: 26.95% (baseline), 26.95% (FU); OR: 1.00 95%CI (0.49–2.04), p>0.05  1. I/C odds at FU: OR=11.97, 95%CI (7.05-20.30), p<0.001. | - Social cohesion: Small villages achieved targets faster. - Age: Children may attend areas that are not ODF. - Occupation (-): Lack of options if working in the open rural settings. - Socio-cultural (-): gender and religion. - Infrastructure (-): Absence of public infrastructure in school and work areas, transmission may continue outside HHs. - Time (-): BC benefits may happen in the long term. |
|  |  |  |  |  |  | Epidemiological: | 1. Perc. of children and adults with positive diagnosis (All Schistosomiasis).^(g)^  - All SCH - Intervention: 0.94% (baseline), 0.24% (FU), p>0.05 - All SCH - Control: 15.58% (baseline), 5.71% (FU), p<0.05 - I/C Overall SCH prevalence at FU: OR=0.24 95%CI (0.10-0.59), p=0.002  1. Perc. of children and adults with positive S. Mansoni diagnosis ^(g)^  - S. mansoni – Intervention: 0.94% (baseline), 0.71% (FU), p>0.05 - S. mansoni – Control: 1.04% (baseline), 1.04% (FU), p>0.05 - I/C S. mansoni at FU: OR=0.68, 95%CI(0.15-3.04), p=0.61.  1. Perc. of children and adults with positive S. Haematobium diagnosis ^(g)^  - S. haematobium – Intervention: 0% (baseline), 0.71% (FU), p>0.05 - S. haematobium – Control: 14.81% (baseline), 4.68% (FU), p<0.05 - I/C S haematobium prevalence at FU: OR=0.15, 95%CI(0.04-0.50), p<0.01. |  |
| Jia Gang et al., 2005 [13] | - Dec 1998 – Feb 2000 | - Pre-post measures - Non-equivalent groups. - Non-random assignation. | - Control villages participated of regular MDA campaigns. | - Treatment records - Parasitological surveys | - N-intervention = 423 - N-control =362 | Treatment seeking / uptake | 1. Percentage of villagers treated with PZQ (observed) **^(f)^**:  - Intervention: 28.6% (baseline), 29.1% (FU) - Control: 97.5% (baseline), 95.0% (FU).  1. Percentage of egg positive villagers for S japonicum treated (observed) **^(h)^**  - Intervention: 96.2% (baseline), 97.1% (FU) - Control: 100%(baseline), 100% (FU) | - History of public interventions: A decade of successful MDAs. - Public health systems: Strong and supportive PHC system. - Community resources: Local resources available to the project. - Governance: strong support from local authorities. |
|  |  |  |  |  |  | Epidemiological | 1. Percentage of villagers with positive S. japonicum diagnosis **^(f)^**:  - Intervention: 12.3% (baseline), 8.0% (FU) - Control: 11.0% (baseline), 6.4% (FU) |  |
| Knopp et al., 2019 [14] | - Nov 2011 – Dec 2017 | - Pre-post measures - Equivalent groups. - Random assignation. | - Arm 1: biannual MDA with PZQ. - Arm 2: biannual MDA with PZQ and snail control. - Arms 1 and 2: No teaching programme, safe-games, day events, or infrastructure | - Review of records - Parasitological survey | 1. Intervention (Arm 3):  - n-schools = 30 - n- schoolchildren (9-12) = 2638  1. Arm 1:  - n-schools: 30 - n-schoolchildren (9-12) = 2899  1. Arm 2:  - n-schools = 29 - n- schoolchildren = 2741 | Transmission | N.R. | - Time: BC benefits may happen in the long term. - Infrastructure: Limited child-friendly sanitation infrastructure. |
|  |  |  |  |  |  | Treatment seeking / uptake | 1. Prop. of schoolchildren (6-12) that received treatment (observed): **^(h)^**  - Arm 1: 73.5% (Round 4), 97.9% (Round 10) - Arm 2: 69.8% (Round 4), 97.6% (Round 10) - Intervention: 80.1% (Round 4), 98.1% (Round 10) |  |
|  |  |  |  |  |  | Epidemiological | 1. Prop. of schoolchildren with positive S. haematobium diagnosis (observed):  - Arm 1: 4.2% (baseline),1.4% (Year 6 FU) - Arm 2: 7.8% (baseline), 1.7% (Year 6 FU) - Arm 3: 6.47% (baseline), 1.9% (Year 6 FU) - Arm 2 / Arm 1 odds of positive diagnosis: AOR=1.19, 95%CI(0.5-2.6), p=0.66 - Arm 3 / Arm 1 odds of positive diagnosis: AOR=1.44, 95%CI(0.7-3.1), p=0.38.  1. Prop. of schoolchildren with heavy intensity infections (observed):  - Arm 1: 0.9% (baseline), 0.4% (Year 6 FU) - Arm 2: 1.8% (baseline), 0.3% (Year 6 FU) - Arm 3 : 2.0% (baseline), 0.4% (Year 6 FU) - Arm 2 / Arm 1 odds of heavy intensity infections: AOR=0.95, 95%CI(0.3-3.33), p=0.94 - Arm 3 / Arm 1 odds of heavy intensity infections: AOR=1.80, 95%CI(0.6-6.0), p=0.32. |  |
| Kosinski, et al., 2016 [15] | - June 2008-Aug 2010 | - Pre-post measures - Non-equivalent control. - Non-random assignation. | - Comparisons across cohorts - 2008 cohort: school-enrolled pupils (>8) screened 3+ times. - 2009 & 2010 cohorts: school-enrolled (>8), screened 3+ times and treated with PZQ. - 2009 & 2010 (Previous Infection Status Known): school-enrolled (>8), screened 3+ times, treated with PZQ, and positive infection status known. |  | - n-2008 = 247 - n-2009 = 216 - n-2009 PISK = 133 - n-2010 = 245 - n-2010 PISK = 186 | Exposure | 1. Perc. of schoolchildren using local river (2009 observation):   - 2008 cohort: 10.2%  - 2009 cohort: 12.5%  - 2010 cohort: 13.3% | - N.R. |
|  |  |  |  |  |  | Epidemiological | 1. Perc. of schoolchildren with positive *S. haematobium* diagnosis.  - Girls: 34.2% (baseline), 11.5% (2009), 1.9% (2010) - Boys: 50.0% (2008), 15.0% (2009), 5.0% (2010) - Incidence comparisons in the absence of the WRA (08-09) against with WRA (2009-10): x^2^ =7.57, p=0.01 (Girls); x^2^= 7.43, p=0.01 (Boys). |  |
| Lansdown et al 2002 [16] | - Mar 1998 – April 2000 | - Pre-post measures - Non-equivalent groups. - Random assignation. | - No health education | - KAP survey - Direct observations | a. Intervention (KAP):   - n-schools= 25 - n-schoolchildren (7-15) = 168 (BL)   b. Control (KAP):   - n-schools = 25 - n-schoolchildren (7-15) = 112 (BL)   c. Intervention and Control (KAP)l:   - n-schoolchildren = 196 (9-month FU)   d. Intervention and Control (Observations):   - n-schools = 6 (9-month FU), 3 (15-month FU) | Exposure | 1. No. of schools that made boiled water available – Observed ^(b)^:   - Intervention 6 (FU1), 3 (FU2)  - Control: 0 (FU1), 0 (FU2)   1. No. of schools that provided water for hand-washing ^(b)^:   - Intervention 6 (FU1), 3 (FU2)  - Control: 0 (FU1), 0 (FU2) | - Interpersonal relations: children have little agency to change adults’ preferences / requests. - Teachers’ leadership - Community resources: School improvements requested community material contributions. - Public programmes support: Cooperations between MoE and the MoHs’ school health programme enabled actions. |
|  |  |  |  |  |  | Transmission | c. No. of schools with evidence of open defaecation  - Intervention: 0 (FU1), 0 (FU2)  - Control: 4 (FU1), 0 (FU2)  d. No. of schools that had latrines clean  - Intervention 5 (FU1), 3 (FU2)  - Control: 3 (FU1), 3 (FU2)  e. No. of schools that provided urinals  - Intervention 3 (FU1), 2 (FU2)  - Control: 3 (FU1), 1 (FU2) |  |
| Madon et al. 2018 [17] | - February 2015 - April 2016. | - Pre-post measures - Equivalent control - Random assignation | - Control group received no intervention of any kind. | - School surveys - Household surveys | a. Intervention:  - N villages = 4  - N villagers = 934 (baseline), 870 (FU).  - N schools = 4  - N schoolchildren= 894 (baseline), 945 (FU).  b. Control:  - N villages = 4  - N villagers = 770 (baseline), 844 (FU)  - N schools = 4  - N schoolchildren= 810 (baseline), 833 (FU). | Transmission. | 1. Perc. of schoolchildren using latrine as family defecation location:  - Intervention: 97.8% (baseline), 98.6% (FU); OR (FU/baseline)=1.66, p>0.05 - Control: 98.9% (baseline), 97.4% (FU); OR (FU/baseline)=0.41, p<0.05. - I/C Ratio of OR – Latrine as family defecation location: ROR=4.01, 95%CI(1.23–13.07), p<0.05.  1. Perc. of households without faeces present around compound  - Intervention: 7.8% (baseline), 6.3% (FU); OR (FU/baseline)=0.84, p>0.05. - Control: 11.0% (baseline), 0.73% (FU); OR (FU/baseline)=0.06, p<0.05. - I/C Ratio of OR – Faeces present around compound: ROR=13.84, 95%CI(1.53–125.12), p<0.05  1. Prop. of households with latrine’s drop-hole covered  - Intervention: 38.0% (baseline), 47.7% (post); OR (post/baseline)=1.44, p>0.05. - Control: 56.8% (baseline), 38.0% (post); OR (post/baseline)=0.49, p<0.05. - I/C Ratio of OR - Drop hole covered: ROR=2.93, 95%CI(1.27–6.88), p<0.05 | - Governance: Fines / sanctions are contentious. - Governance: Limited financial transparency / accountability. - Community resources: Unclear sustainability due to local need for financial and professional support. - Public programme’s engagement: Long-term progress may rely on integration with public health systems. |
|  |  |  |  |  |  | Epidemiological | 1. Perc. of pupils with SCH (any) (self-reported):  - Intervention: 11.4% (baseline), 7.0% (FU); OR (FU/baseline)=0.55, p<0.05. - Control: 8.1% (baseline), 7.0% (FU); OR (FU/baseline)=0.83, p>0.05. - I/C Ratio of OR – All SCH: ROR=0.67, 95% CI(0.48-0.93), p<0.05  1. Perc. of schoolchildren positive for S. Mansoni (self-reported)  - Intervention: 2.7% (baseline), 1.6% (FU); OR (FU/baseline)=0.56, p<0.05. - Control: 1.6% (baseline), 2.4% (FU); OR (FU/baseline)=1.49, p>0.05. - I/C Ratio of OR – S. Mansoni: ROR=0.37, 95%CI(0.22-0.64), p<0.05.  1. Perc. of schoolchildren positive for S. Haematobium (self-reported)  - Intervention: 10.5% (baseline), 6.3% (FU); OR (FU/baseline)=0.55, p <0.05. - Control: 7.0% (baseline), 6.2% (FU); OR (FU /baseline)=0.87, p <0.05. - I/C Ratio of OR – S. haematobium: ROR=0.63 95%CI(0.44-0.91), p<0.05 |  |
| Muhumuza et al., 2014 [18] | - May- July 2013 | - Pre-post - Equivalent groups - Random assignation | - MDA with praziquantel - Health education - No snack provision | - KAP survey - Parasitological survey | a. Intervention:   - n-schools=12 - n-pupils=620   Control:   - n-schools= 12 - n-pupils: 696 | a. Health seeking behaviour: | a.. Perc. of pupils who received treatment (self-reported):   - Intervention: 46.5% (baseline), 93.9% (FU) (95% CI 91.7%–95.7%) - Control: 51.0% (baseline), 78.7% (FU) (95% CI 75.4%–81.7%). - X^2^ (baseline) =0.322, p=0.57 - X^2^ (FU) =9.683, p=0.002 | N.R. |
|  |  |  |  |  |  | a. Epidemiological | a. Percentage of pupils with positive S.  mansoni diagnosis: ^(i)^   - Intervention: 34.4% (baseline), 1.3% (FU) (95% CI 0.6%–2.6%) - Control: 30.9% (baseline), 14.1% (FU) (95% CI 11.6%–16.9% - X^2^ (baseline)=0.217, p=0.64 - X^2^ (FU) =10.937, p=0.001   b. Average eggs per gram of stool:   - Intervention: 115.9 (baseline), 38.3 (FU) (95% CI 21.8–67.2) - Control: 120.5 (baseline), 78.4 (FU) (95% CI 60.6–101.5) - t test (baseline) =.0126, p=0.26   t-test (FU) t=19.54, p=0.001 |  |
| Mwanga et al., 2013 [19] | - 2008-2010 | - Pre-post measures - Single group - Non-random assignation | - N.A. | - KAP survey - Direct observation | - N-villages=1 - N-households=200 - N-adults (baseline)=216 - N-adults (FU – Year 3)=157 (matched). - N-observation sites= 5 (12 days). | Exposure | a. Perc. of men bathing in lake (self-reported), p>0.05 **^(f)^**  b. Perc. of women bathing in lake (self-reported): 78% (baseline), 63% (FU), p=0.005  c. Perc. of men fetching water from lake (self-reported): 82% (baseline), 50% (FU), p = 0.004.  d. Perc. of women fetching water from lake (self-reported): 66.5% (baseline), 53.5% (FU), p = 0.02.  e. Perc. of all water-contact events that were conducted by male residents: 36% (baseline), 33% (FU), p<0.001  f. Perc. of all water-contact events that were conducted by female residents: 64% (baseline), 67% (FU), p<0.001  g. Perc. of all water-contact events that were conducted by residents 0-4 (observed): 4.9% (baseline), 4% (FU), p<0.01  h. Perc. of all water-contact events that were conducted by residents 5-14 (observed): 30.9% (baseline), 28% (FU), p<0.01  i. Perc. of all water-contact events that were conducted by residents 15-64 (observed): 64.2% (baseline), 68% (FU), p<0.01  j. Average time per water contact event by male residents: 10.8m (baseline), 8.8m (FU), p<0.01  k. Average time per water contact event by female residents: 7.4m (baseline), 5.7m (FU), p<0.01  l. Average time per water contact event by residents 0-4: 8.4m (baseline), 5.8m (FU), p<0.01  m. Average time per water contact event by residents 5-14 (observed): 8.6m (baseline), 7.1m (FU), p<0.01  m. Average time per water contact event by residents >15 (observed): 8.6m (baseline), 6.6m (FU), p<0.01 | - Socio-cultural (-): Gender-roles shaped participation and exposure. - Socio-cultural (-): Age cohorts shaped exposure. - Infrastructure (-): Lack of alternative safe water sources. |
|  |  |  |  |  |  | Treatment uptake / seeking | a. Perc. of men seeking treatment for S mansoni: p>0.05**^(f)^**.  b. Perc. of women seeking treatment for S mansoni: 4.8% (baseline),13.4% (FU), p<0.001. |  |
| Mwanga et al., 2015 [20] | - 2009-2012 | - Pre-post measures - Single group - Non-random assignation | - N.A. | KAP surveys | - n-villages= 1 - n-adults =82 (BL and FU, matched) | Exposure | a. Perc. of adults that avoided contact with infected water: 12.2% (BL), 81.7% (FU), p<0.01  b. Perc. of adults that avoided contact with dirty water: 6.1% (baseline), 75.6% (FU), p<0.01  c. Perc. of adults that only used /use safe water sources: 3.7% (baseline), 31.7% (FU), p<0.01  d. Perc. of adults that bathed at home: 11.0% (baseline), 52.4% (FU), p<0.01 | - Infrastructure: Provision of pumped wells enhanced outcomes. - Socio-cultural (-): Engagement varied by gender. |
| N’Diaye et al., 2016 [21] | - 2009-2015 | - Pre-post measures - Single group - Non-random assignation. | - N.A. | Parasitological surveys | 1. School aged children (SAC) (6-14):  - n-villages = 53 - n-SAC (2009) =1108 - n-SAC (2010) =1108 - n-SAC (2011) =1108 - n-SAC (2012) =1225 - n-SAC (2013) =1047 - n-SAC (2014) =1142  1. Pre-SAC (0-5):   - n-villages =1   - n-Pre-SAC (2008) = 82 - n-Pre-SAC (2009) = 61 - n-Pre-SAC (2011) = 88 - n-Pre-SAC (2014) = 83 - n-Pre-SAC (2015) = 108 | Exposure | N.R. | - Infrastructure: lack of safe water sources. - Infrastructure: lack of safe public footpaths. - Environment: marshlands - Occupation: fishing. |
|  |  |  |  |  |  | Transmission | N.R. |  |
|  |  |  |  |  |  | Epidemiological: | a. Perc. of SAC with positive *S. mansoni* diagnosis (observed)^(f)^. SAC: 44% (baseline), 1.35% (2009), <5% since then until 2014.  b. Perc. of SAC with positive *S. haematobium* diagnosis (observed)^(f)^ : <5% at baseline, <10% since treatment year until end of intervention. (*)  c. Perc. of PSAC with positive *S. Mansoni* diagnosis (observed) ^(f)^: 78% (2008), 59% (2009), 47.4% (2011a), 18.2% (2011b), 9.6% (2014), 12.9% (2015). |  |
| Nagi et al. 2005 [22] | 1999-2001 ^(a)^ | - Pre-post measures - Single group - Non-random allocation | - N.A. | KAP surveys | a. Community survey   - n-households = 100 - n-villagers = 863 (BL), 913 (FU)   b. School survey:   - n-schools = 14 - n-schoolchildren = 287 (BL), 323 (FU) | Exposure | 1. Percentage of SAC and adults reporting contact with water source (self-reported): 95% (baseline), 9% (FU)^(f)^ 2. Percentage of school-enrolled SAC reporting contact with water source (self-reported): 98% (baseline), 3.6% (FU)^(f)^ 3. Percentage of SAC and adults adopting preventive measures (self-reported): 0.9% (baseline), 97% (FU)^(f)^ 4. Percentage of school-enrolled SAC adopting preventive measures (self-reported): 0% (baseline), 88% (FU)^(f)^ | - School enrolment |
|  |  |  |  |  |  | Treatment seeking / uptake | 1. Percentage of SAC and adults that received treatment (self-reported):: 0% (baseline, no treatment), 95% (FU)**^(f)^** 2. Percentage of school-enrolled SAC that received treatment (self-reported):: 0% (baseline, no treatment), 97.3% (FU) **^(f)^** |  |
|  |  |  |  |  |  | Epidemiological: | 1. Percentage of SAC and adults with positive S. haematobium diagnosis (observed): 58.9% (baseline), 5.8% (FU)**^(f)^** 2. Percentage of infected SAC and adults with heavy S. haematobium infection (observed): 40% (baseline), 18.9% (FU) **^(f)^** 3. Percentage of school-enrolled SAC with positive S. haematobium diagnosis (observed): 69.9% (baseline), 4.9% (FU) **^(f)^** 4. Percentage of infected school-enrolled SAC with heavy S. haematobium infection (observed): 38% (baseline), 16.7% (FU) **^(f)^** |  |
| Noda et al., 1997 [23] | - Jun 1982- May 1986 | - Pre-post measures - Single group - Non-random assignation | - N.A. | - Direct observation - Standpipe use records | - n-residents observed (average) = 179 (BL), 114 (FU) | Exposure | 1. No. of people conducting water contact activities:   - Males: 179 (BL), 114 (FU), p<0.05  - Females: 177 (BL), 117 (FU), p<0.05   1. Total number of water contact activities   - Males: 395 (BL), 208 (FU), p<0.05  - Females: 442 (BL), 260(FU), p<0.05   1. Average number of water contact activities per person   - Males: 1.74 (BL), 1.53 (FU), p<0.05  - Females: 1.89 (BL), 1.76(FU), p<0.05   1. Total amount of time spent in water contact activities   - Males: 4668(BL), 3139 (FU), p<0.05  - Females: 4191 (BL), 3470(FU), p>0.05   1. Average amount of time spent in water contact activities per person   - Males: 5.30 (BL), 6.89(FU), p>0.05  - Females: 5.39 (BL), 7.09(FU), p>0.05   1. No. of water contact activities for washing clothes in the riverbank   - Males: 20.0 (BL), 10.9 (FU), p<0.05  - Females: 55.6 (BL), 34.4 (FU), p<0.05   1. No. of water contact activities for washing clothes on the riverbank   - Males: 4.6 (BL), 7.3 (FU), p>0.05  - Females: 19.2 (BL), 30.3 (FU), p>0.05   1. No. of water contact activities for washing utensils   - Males: 2.6 (BL), 2.6 (FU), p>0.05  - Females: 19.8 (BL), 19.3 (FU), p>0.05   1. No. of water contact activities for bathing   - Males: 178.8 (BL), 111.4 (FU), p<0.05  - Females: 164.6 (BL), 115.9 (FU), p<0.05   1. No. of water contact activities for washing the body   - Males: 52.2 (BL), 21.6 (FU), p<0.05  - Females: 87.2 (BL), 36.4 (FU), p<0.05   1. No. of water contact activities for collection of water   - Males: 13.6 (BL), 15.3 (FU), p>0.05  - Females: 199.0 (BL), 133.1 (FU), p<0.05   1. No. of water contact activities for playing   - Males: 34.0 (BL), 20.3 (FU), p<0.05  - Females: 22.0 (BL), 8.3 (FU), p<0.05   1. No. of water contact activities for fishing   - Males: 47.0 (BL), 31.9 (FU), p>0.05  - Females: 19.8 (BL), 154 (FU), p>0.05 | - Infrastructure: Limited number of standpipes. - Infrastructure: lack of washing stations or distribution mechanisms. - Rurality: population dispersion. - Costs: financial payments for use / maintenance. - Gender norms |
| Nsowah-Nuamah et al. 2001 [24] | - 1993-1997 ^(a)^ | - Pre-post measures - Non-equivalent groups - Non-random assignation | - Arm 1: Passive health education and treatment - Arm 2: No health education and treatment - Arm 3: Active health education and treatment | - Direct observation - Parasitological survey | a. Arm 1:  n-villages=2  n-villagers=697 (BL), 784 (24m follow up)  b. Arm 2:  n-villages=2  n-villagers= 775 (BL), 713 (24-m follow up)  c. Arm 3:  n-villages=4  n-villagers= 1090 (BL), 1075(24m follow-up) | Exposure | 1. No. of wells built   - Arm 1: 3 (BL), 4 (FU)  - Arm 2: 0 (BL), 1 (FU)  - Arm 3: 0 (BL), 8 (FU)   1. No. piped-water installations built   - Arm 1: 0 (BL), 0 (FU)  - Arm 2: 8 (BL), 8 (FU)  - Arm 3: 0 (BL), 0 (FU) | - Sex - Gender norms - Occupation - Community resources to complete all facilities to the desired state (e.g., pumps). - Environment: suitable subterranean water sources. - Infrastructure: Additional public infrastructure needed (safe water) - Time: Need to sustain messages (long-term, like through curricula). - Community governance: Local organisations needed to show capacity / initiative to reach regional development orgs. |
|  |  |  |  |  |  | Transmission | 1. No. of school toilets built   - Arm 1: 1 (BL), 1 (FU)  - Arm 2: 0 (BL), 0 (FU)  - Arm 3: 0 (BL), 2 (FU)   1. No. of public latrines built.   - Arm 1: 8 (BL), 8 (FU)  - Arm 2: 0 (BL), 0 (FU)  - Arm 3: 10 (BL), 13 (FU)   1. No. of private latrines built   - Arm 1: 14 (BL), 14 (FU)  - Arm 2: 6 (BL), 7 (FU)  - Arm 3: 8 (BL), 23 (FU) |  |
|  |  |  |  |  |  | Epidemiological | 1. Per. of residents (>5) with positive diagnostic for *S. haematobium*  - Area 1-male: 62.3% (BL), 20.3% (FU) - Area 2-male: 67.3% (BL), 23.7% (FU) - Area 3-male: 63.2% (BL), 40.8% (FU) - Area 1-female: 50.9% (BL), 16.7% (FU) - Area 2-female: 29.7% (BL), 14.4% (FU) - Area 3-female: 47.0% (BL), 31.2% (FU) - Area 3 / Area 1 comparison post intervention (Logit B coefficient on likelihood of infection): -1.893, p<0.01 - Area 3 / Area 2 comparison post intervention (Logit B coefficient on likelihood of infection): -1.434, p<0.01  1. Per. of residents (>5) positive for *S. haematobium* with heavy intensity infection  - Area 1: 43.9% (BL), 28.5% (FU) - Area 2: 49.3% (BL), 24.2% (FU) - Area 3: 49.3% (BL), 39.8% (FU) - Area 3 / Area 1 comparison post intervention (Gen. linear B coefficient on intensity of infection): -64.840, p=0.175 - Area 3 / Area 2 comparison post intervention (Gen. linear B coefficient on intensity of infection): 67.899, p=0.028 |  |
| Oyeyemi et al. 2018 [25] | - Jun 2015 – Jan 2017 | - Pre-post measures - Single group - Non-random assignation | - N.A. | - Parasitological survey | - n-village=1 - n-villagers (3-86) =77 | Exposure | N.R. | - Infrastructure: Borehole provision essential but insufficient. Additional WASH infrastructure required. |
|  |  |  |  |  |  | Epidemiological | 1. Perc. of villagers (-3-86) with positive SCH diagnosis. 20.8% (baseline), 2.6% (FU), p<0.05 2. Average number of eggs per 10ml urine among positive cases (3-86): 19.3 (baseline), 1.4 (FU), p<0.05 |  |
| Person et al., 2021 [26] | February 2017 | - Post measures - Equivalent groups. - Random assignation. | - Arm 1: biannual MDA with PZQ. - Arm 2: biannual MDA with PZQ and snail control. - Arms 1 and 2: No teaching programme, safe-games, day events, or infrastructure | - KAP survey | 1. Intervention (Arm 3):  - n-schools: 8 - n-schoolchildren (9-16) = 708  1. Control (Arms 1 and 2)  - n-schools = 4   n-schoolchildren (9-16) = 743 | Exposure | 1. Perc. of children that spend less time washing at stream/pond (self-reported)  - Pemba girls: 13.2% intervention, 0% control (p<0.001) - Pemba boys: 23.6% intervention, 2.6% control (p<0.001) - Unguja girls: 3.6% intervention, 0% control (p=0.005) - Unguja boys: 4.8% intervention, 0.6% control (p=0.028)  1. Perc. of schoolchildren that stopped washing at stream/pond (self-reported)  - Pemba girls: 61.8% intervention, 0% control (p<0.001) - Pemba boys: 36.0% intervention, 5.8% control (p<0.001) - Unguja girls: 59.5% intervention, 2.7% control (p<0.001) - Unguja boys: 48.4% intervention, 3.1% control (p<0.001)  1. Perc. of schoolchildren that spend less time bathing at stream/pond (self-reported)  - Pemba girls: 1.8% intervention, 0% control (p=0.007) - Pemba boys: 0.6% intervention, 1.3% control (p=0.486) - Unguja girls: 15.4% intervention, 0.5% control (p<0.001) - Unguja boys: 17.7% intervention, 3.1% control (p<0.001)  1. Perc. of schoolchildren that stopped bathing at stream/pond (self-reported)  - Pemba girls: 51.3% intervention, 0% control (p<0.001) - Pemba boys: 35.4% intervention, 12.9% control (p<0.001) - Unguja girls: 50.8% intervention, 10.4% control (p<0.001) - Unguja boys: 41.9% intervention, 6.8% control (p<0.001)  1. Perc. of schoolchildren that report spending less time swimming/playing at stream/pond  - Pemba girls: 2.2% intervention, 0.5% control (p=0.136) - Pemba boys: 1.9% intervention, 9.7% control (p=0.002) - Unguja girls: 3.6% intervention, 1.4% control (p=0.121) - Unguja boys: 1.6% intervention, 4.9% control (p=0.115)  1. Perc. of schoolchildren that reported having stopped swimming/playing at stream/pond  - Pemba girls: 45.6% intervention, 0% control (p<0.001) - Pemba boys: 46.0% intervention, 40.7% control (p=0.200) - Unguja girls: 33.9% intervention, 3.6% control (p<0.001) - Unguja boys: 36.3% intervention, 5.6% control (p<0.001) | - Socio-cultural: traditional understandings of disease. - Social influence: adults’ views affect children’s actions. - Cross-contamination: Teaching materials were shared in some schools from control group. |
|  |  |  |  |  |  | Transmission | - N.R. |  |
|  |  |  |  |  |  | Health-seeking / Treatment Uptake | 1. Perc. of schoolchildren that now swallow PZQ tablets when not before (self-reported)  - Pemba girls: 49.6% intervention, 0% control (p<0.001) - Pemba boys: 40.4% intervention, 21.9% control (p<0.001) - Unguja girls: 12.3% intervention, 0% control (p<0.001) - Unguja boys: 9.7% intervention, 0% control (p<0.001) |  |
| Rassi et al., 2019 [27] | - July 2014-Dec 2015 | - Pre-post measures - Single group - Non-random assignation | - N.A. |  | - n-districts=4 - n-adults =791 (BL), 792 (FU) | Exposure | 1. Perc. of adults who avoid swimming in infested water(self-reported): 26.58% (95% CI: 19.79% -34.68%) (baseline), 46.90% (95%CI: 39.00% - 54.96%). 2. Perc. of adults who report boiling bathing water (self-reported): 6.76% (95%CI: 3.61% - 12.29%) (baseline), 4.65% (95%CI: 2.92% - 7.32%) | - Occupation - Socio-cultural: risk practices embedded in traditions and norms. - Community resources: lack of skills and resources for technical solutions. - Infrastructure: Lack of latrine investments. - Time: BC is long-term process. |
|  |  |  |  |  |  | Transmission behaviour: | 1. Perc of adults that used latrines (self-reported): 18.02% (95%CI 12.42% - 25.41%) (baseline), 17.83% (95%CI: 13.32% - 23.46%) (FU). |  |
|  |  |  |  |  |  | Treatment seeking / uptake | 1. Perc. of adults with minors living in household where at least one of them received (self-reported): PZQ: 9.33% (baseline), 95%CI(6.69-12.86); 15.10% (FU), 95%CI (11.30%-19.89%); AOR (FU/baseline) =1.62, 95%CI(1.15-2.28), p<0.01 2. Perc. of adults with minors living in household who would like their children receive PZQ (self-reported): 93.33% (baseline), 95%CI (90.33%-95.45%); 87.12% (FU), 95%CI (83.67%-89.94%); AOR (FU/baseline) = 0.44, 95%CI (0.27-0.70), p<0.01 |  |
| Stothard et al., 2016 [28] | - Dec. 2005 – Jan. 2007 | - Pre-post measures - Single group - Non-random assignation | - N.A. | - KAP survey | - n- schoolchildren = 751 (BL), 779 (FU) | Exposure | 1. Perc. of schoolchildren reporting water contact activities (playing, working or washing): range: 15%-20% for baseline and FU | - Infrastructure: lack of safe water areas. - Socio-cultural traditions: recreational practices - Occupation: Fishing. |
|  |  |  |  |  |  | Transmission | 1. Perc. of schoolchildren urinating in water sources (self-reported): 14.8% (baseline) (95% CI: 13.3%-16.4%), 5.7% (FU) (95% CI: 4.9%-6.5%), z= 10.91, p< 0.001 [29] |  |
| Wang et al., 2013 [30] | - June 2007 – Oct 2009 | - Pre-post measures - Equivalent control - Random assignation | - No health education | - KAP survey | a. Intervention   - n-villages = 13 villages - n-individuals (baseline) = 358 - n-individuals (FU 1) = 131 - n-individuals (FU 2) = 229   b. Control   - n-villages = 13 villages - n-individuals (baseline) = 348 - n-individuals (FU 1) = 77 - n-individuals (FU 2) = 212 | Exposure | 1. Perc. of adults with unprotected water contact  - Intervention: 88.6% (baseline), FU1 and FU2 N.R. - Control: 85.5% (baseline), FU1 and FU2 N.R. - Adjusted I/C OR on FU1-FU2 differences =1.15, 95%CI (0.55, 2.43).  1. Adult’s average PPE wearing behaviour score:  - Intervention: 30.2 (baseline), 14.1 (FU1); 30.4 (FU2). - Control: 28.8 (baseline), 14.1 (FU1), 24.9 (FU2). - Adjusted I-C group differences: 1.74, 95%CI(−6.12, 9.60) (baseline); 1.78, 95%CI(−4.18, 7.74) (FU1); 4.42, 95%CI(−3.38, 12.22) (FU2).* | - Occupation: farming. - Infrastructure: Lack of adequate WASH infrastructure. - Practicalities: PPE cumbersome for work. - Environment (-): topography limits technification, manual work needed. - Costs: Defaecate away from fields has few barriers. - Cross-contamination (-): Novelty of intervention led to information sharing (cross-contamination). |
|  |  |  |  |  |  | Transmission | 1. Perc. of adults conducting open defecation (self-reported)  - Intervention: 17.0% (baseline), 8.1% (FU1), 2.4% (FU2) - Control: 18.7 (baseline), 10.3% (FU1); 7.0% (FU2) - Adjusted I-C group differences: 1.54 (baseline), 95%CI (0.45, 5.24); 1.21 (FU 1), 95%CI (0.32, 4.53); 3.28 (FU 2), 95%CI (0.57, 18.98)* |  |
|  |  |  |  |  |  | Treatment seeking / uptake | 1. Adults’ average attitudinal score for index on infection testing and treatment  - Intervention: 82.6 (baseline), 84.5 (FU1), 91.0 (FU2); - Control: 81.2 (baseline), 82.5 (FU1), 87.3 (FU 2) - Adjusted I-C differences: 2.26, 95%CI (−2.68, 7.19) (baseline); 1.55, 95%CI (−4.20, 7.29) (FU1); 3.65, 95%CI (−1.75, 9.05) (FU2) *   (*) Comparisons are between regression scores using generalized estimating equations |  |
| Wepnje et al., 2019 [31] | - Nov 2016 - Jan 2018 | - Pre-post measures - Single group - Non-random assignation | - N.A. | - KAP survey - Parasitological survey | - n-village = 1 - n - pregnant women = 368 | Exposure | a. Per. of pregnant women reporting using stream water   - 99.2% (baseline), 76.1% (FU) - Absolute Risk Reduction: 0.23, 95%CI (0.19 – 0.28)   b. Perc. of pregnant women frequenting stream 3 or more times per week   - 51.2% (baseline), 15.7% (FU) - Absolute Risk Reduction: 0.23, 95%CI (0.28 – 0.43)   c. Perc. of pregnant women using stream for domestic activities and bathing   - 50.4% (baseline), 31.8% (FU) - Absolute Risk Reduction: 0.23, 95%CI (0.10 – 0.27)   d. Perc. of pregnant women using stream for domestic activities only   - 49.6% (baseline), 68.2% (FU) - Absolute Risk Reduction: N.A. | - Education: less likelihood of infection - Marital status: HH roles. - Infrastructure: Additional standpipes required. - Rurality: Population dispersion. - Costs: Payment to access piped water |
|  |  |  |  |  |  | Epidemiological | e. Perc. of pregnant women with positive S. haematobium diagnosis   - 46.8% (baseline), 22.3% (FU) - Absolute Risk Reduction: 0.25, 95%CI (0.17– 0.32) |  |
|  |  |  |  |  |  |  | f. Per. of pregnant women with heavy intensity infection   - 21.2% (baseline), 6.3% (FU) - Absolute Risk Reduction: 0.15, 95%CI (0.10 – 0.32) |  |
| Wolmarans & de Knock, 2009 [32] | - Jan- 2004 - Dec. 2006 | - Pre-post measures - Equivalent group - Non-random assignation | - Two control groups from same school - Untreated control: no health education nor treatment. - Treated control: treatment but no health education. | Parasitological surveys | a. Intervention:   - n-schools =1 - n-schoolchildren (infected) = 67   b. Control - Untreated:   - n-schools =1 - n- schoolchildren (infected) = 80   c. Control - Treated:   - n-schools =1 - n- schoolchildren (infected)= 99 | Exposure | - N.R. |  |
|  |  |  |  |  |  | Epidemiological: | a. Perc. of schoolchildren with positive S. haematobium diagnosis (observed) **^(f)^**   - Intervention: >75% (baseline), <10% (FU2 - FU7). - Untreated control: >75% (baseline), no significant change across 7 follow-ups. - Treated control: >75% (baseline), <25% (FU1), 40% - 60% (FU 2 and FU 5), 25% at FU 7.   b. Prop. of schoolchildren with heavy intensity infections   - Intervention: 20.8% (baseline),1.9% (FU 7). - Untreated control: 6.4% (baseline), 17.6% (FU7). - Treated control: 17.1% (baseline), 0 % (FU 7). - Mann-Whitney U Test: p<0.05, except for experimental and treated control (p=0.84)   c. Prop. of infected schoolchildren (4-14) with haematuria   - Intervention: Baseline >80%, <20% FU 1 – FU3, <10% since F5. - Untreated control: Baseline >70%, >70% FU 1 - FU4, >60% FU 5- FU 7. - Treated control: : Baseline >90%, 60%-70% since FU 1 until FU 7 - Mann-Whitney U Test: p<0.05, except for experimental and treated control (p=0.57) | - Social networks: Educational activities reached the wider population despite their lack of direct involvement |
| Yuan et al., 2000 [33] | - July 1995 – Sep 1996 | - Pre-post measures - Equivalent groups - Random assignation | - No health education | - KAP surveys - Direct observation | a. Intervention:   - n-schools = 24 - n-schoolchildren = N.R. (BL), 875 (FU)   b. Control:   - n-schools= 24 - n-schoolchildren = N.R., 864 (FU)   c. Intervention and Control:   - n- schoolchildren TOTAL = 2263 (BL) | Exposure | a. Perc. of schoolchildren don’t swim in lake (self-reported)  - Intervention: 46.9% (BL), 67.5% (FU)  - Control: 40.3% (BL), 68.3% (FU)  - X^2^= 3.88, p>0.1  b. Perc. of schoolchildren using safe water sources (self-reported)  - Intervention: 36.1% (BL), 62.3% (FU)  - Control: 47.3% (BL), 31.0% (FU)  - X^2^= 63.03, p<0.01  c. Perc. of water contact activities in unsafe locations (observed) ^(a,b)^  - Intervention: >70% (BL), <55% (FU)  - Control: >70% (BL), 71.4% (FU) | - Environment: many surface water areas in public spaces. - Infrastructure: Lack of safe water sources. - Length: Project was short-term. Changes may not be sustained over time. |
| 1. No further details reported 2. No statistical test reported | | | | | | | | |

**References**

1. Chaula SA, Tarimo DS. Impact of praziquantel mass drug administration campaign on prevalence and intensity of Schistosoma haemamtobium among schoolchildren in Bahi district, Tanzania. Tanzan J Health Res [Internet]. 2014 Mar 5 [cited 2020 Mar 21];16(1). Available from: https://www.ajol.info/index.php/thrb/article/view/96721

2. Cline BL, Hewlett BS. Community-based approach to schistosomiasis control. Acta Trop. 1996 Apr 1;61(2):107–19.

3. Ejike CU, Oluwole AS, Mogaji HO, Adeniran AA, Alabi OM, Ekpo UF. Development and testing of Schisto and Ladders^TM^, an innovative health educational game for control of schistosomiasis in schoolchildren. BMC Res Notes. 2017 Jun 28;10(1):236.

4. Ejike CU, Oluwole AS, Omitola OO, Bayegun AA, Shoneye IY, Akeredolu-Ale BI, et al. Schisto and Ladders version 2: a health educational board game to support compliance with school-based mass drug administration with praziquantel – a pilot study. Int Health. 2021 May 1;13(3):281–90.

5. Kholy HE, Siongok TKA, Koech D, Sturrock RF, Houser H, King CH, et al. Effects of Borehole Wells on Water Utilization in Schistosoma Haematobium Endemic Communities in Coast Province, Kenya. Am J Trop Med Hyg. 1989 Aug 1;41(2):212–9.

6. Favre TC, Massara CL, Beck LCNH, Cabello RKSA, Pieri OS. Adherence to diagnosis followed by selective treatment of schistosomiasis mansoni and related knowledge among schoolchildren in an endemic area of Minas Gerais, Brazil, prior to and after the implementation of educational actions. Parasite Epidemiol Control. 2021 May 1;13:e00208.

7. Fink G, Rockers PC. Financial Incentives, Targeting, and Utilization of Child Health Services: Experimental Evidence from Zambia. Health Econ. 2017;26(10):1307–21.

8. Freeman MC, Clasen T, Brooker SJ, Akoko DO, Rheingans R. The Impact of a School-Based Hygiene, Water Quality and Sanitation Intervention on Soil-Transmitted Helminth Reinfection: A Cluster-Randomized Trial. Am J Trop Med Hyg. 2013 Nov 6;89(5):875–83.

9. GARBA A, ABOUBACAR A, BARKIRE A, VERA C, SELLIN B, CHIPPAUX JP. Impact de la sensibilisation des populations dans la lutte contre la bilharziose urinaire au Niger. Impact Sensib Popul Dans Lutte Contre Bilharziose Urin Au Niger. 2001;11(1):35–42.

10. Guang-Han H, Jia H, Kuang-Yu S, Dan-Dan L, Ju Z, Chun-Li C, et al. The role of health education and health promotion in the control of schistosomiasis: experiences from a 12-year intervention study in the Poyang Lake area. Acta Trop. 2005 Nov 1;96(2):232–41.

11. Hong Q biao, Yang K, Huang Y xin, Sun L ping, Yang G jing, Gao Y, et al. Effectiveness of a comprehensive schistosomiasis japonica control program in Jiangsu province, China, from 2005 to 2008. Acta Trop. 2011 Sep;120 Suppl 1:S151-157.

12. Hürlimann E, Silué KD, Zouzou F, Ouattara M, Schmidlin T, Yapi RB, et al. Effect of an integrated intervention package of preventive chemotherapy, community-led total sanitation and health education on the prevalence of helminth and intestinal protozoa infections in Côte d’Ivoire. Parasit Vectors. 2018 Feb 27;11(1):115.

13. Jia-Gang G, Chun-Li C, Guang-Han H, Han L, Dong L, Rong Z, et al. The role of ‘passive chemotherapy’ plus health education for schistosomiasis control in China during maintenance and consolidation phase. Acta Trop. 2005 Dec;96(2–3):177–83.

14. Knopp S, Person B, Ame SM, Ali SM, Hattendorf J, Juma S, et al. Evaluation of integrated interventions layered on mass drug administration for urogenital schistosomiasis elimination: a cluster-randomised trial. Lancet Glob Health. 2019 Aug 1;7(8):e1118–29.

15. Kosinski KC, Kulinkina AV, Abrah AFA, Adjei MN, Breen KM, Chaudhry HM, et al. A mixed-methods approach to understanding water use and water infrastructure in a schistosomiasis-endemic community: case study of Asamama, Ghana. BMC Public Health. 2016;16:322.

16. Lansdown R, Ledward A, Hall A, Issae W, Yona E, Matulu J, et al. Schistosomiasis, helminth infection and health education in Tanzania: achieving behaviour change in primary schools. Health Educ Res. 2002 Aug 1;17(4):425–33.

17. Madon S, Malecela MN, Mashoto K, Donohue R, Mubyazi G, Michael E. The role of community participation for sustainable integrated neglected tropical diseases and water, sanitation and hygiene intervention programs: A pilot project in Tanzania. Soc Sci Med. 2018 Apr 1;202:28–37.

18. Muhumuza S, Olsen A, Katahoire A, Kiragga AN, Nuwaha F. Effectiveness of a Pre-treatment Snack on the Uptake of Mass Treatment for Schistosomiasis in Uganda: A Cluster Randomized Trial. PLOS Med. 2014 May 13;11(5):e1001640.

19. Mwanga JR, Lwambo NJS. Pre- and post-intervention perceptions and water contact behaviour related to schistosomiasis in north-western Tanzania. Acta Trop. 2013 Nov;128(2):391–8.

20. Mwanga JR, Kaatano GM, Siza JE, Chang SY, Ko Y, Kullaya CM, et al. Improved Perceptions and Practices Related to Schistosomiasis and Intestinal Worm Infections Following PHAST Intervention on Kome Island, North-Western Tanzania. Korean J Parasitol. 2015 Oct;53(5):561–9.

21. N’Diaye M, Dioukhane EM, Ndao B, Diedhiou K, Diawara L, Talla I, et al. Schistosomiasis Sustained Control Program in Ethnic Groups around Ninefescha (Eastern Senegal). Am J Trop Med Hyg. 2016 Sep 7;95(3):614–22.

22. Nagi M a. M. Evaluation of a programme for control of schistosoma haematobium infection in Yemen. East Mediterr Health J Rev Sante Mediterr Orient Al-Majallah Al-Sihhiyah Li-Sharq Al-Mutawassit. 2005 Nov;11(5–6):977–87.

23. Noda S, Shimada M, Muhoho ND, Sato K, Kiliku FBM, Gatika SM, et al. Effect of Piped Water Supply on Human Water Contact Patterns in a Schistosoma haematobium-Endemic Area in Coast Province, Kenya. Am J Trop Med Hyg. 1997 Feb 1;56(2):118–26.

24. Nsowah-Nuamah NN, Mensah G, Aryeetey ME, Wagatsuma Y, Bentil G. Urinary schistosomiasis in southern Ghana: a logistic regression approach to data from a community-based integrated control program. Am J Trop Med Hyg. 2001 Nov 1;65(5):484–90.

25. Oyeyemi O, Olowookere D, Ezekiel C, Oso G, Odaibo A. The impact of chemotherapy, education and community water supply on schistosomiasis control in a Southwestern Nigerian village. Infect Dis Health. 2018 Jun 1;23(2):121–3.

26. Person B, Rollinson D, Ali SM, Mohammed UA, A’kadir FM, Kabole F, et al. Evaluation of a urogenital schistosomiasis behavioural intervention among students from rural schools in Unguja and Pemba islands, Zanzibar. Acta Trop. 2021 Aug 1;220:105960.

27. Rassi C, Kajungu D, Martin S, Arroz J, Tallant J, Beyl CZ de, et al. Have You Heard of Schistosomiasis? Knowledge, Attitudes and Practices in Nampula Province, Mozambique. PLoS Negl Trop Dis. 2016 Mar 4;10(3):e0004504.

28. Stothard JR, Khamis AN, Khamis IS, Neo CHE, Wei I, Rollinson D. HEALTH EDUCATION AND THE CONTROL OF UROGENITAL SCHISTOSOMIASIS: ASSESSING THE IMPACT OF THE <span class="italic">JUMA NA KICHOCHO</span> COMIC-STRIP MEDICAL BOOKLET IN ZANZIBAR. J Biosoc Sci. 2016 Sep;48(S1):S40–55.

29. Stothard JR, French MD, Khamis IS, Basáñez MG, Rollinson D. The epidemiology and control of urinary schistosomiasis and soil-transmitted helminthiasis in schoolchildren on Unguja Island, Zanzibar. Trans R Soc Trop Med Hyg. 2009 Oct;103(10):1031–44.

30. Wang S, Carlton EJ, Chen L, Liu Y, Spear RC. Evaluation of an educational intervention on villagers’ knowledge, attitude and behaviour regarding transmission of Schistosoma japonicum in Sichuan province, China. Acta Trop. 2013 Sep 1;127(3):226–35.

31. Wepnje GB, Anchang-Kimbi JK, Ndassi VD, Lehman LG, Kimbi HK. Schistosoma haematobium infection status and its associated risk factors among pregnant women in Munyenge, South West Region, Cameroon following scale-up of communal piped water sources from 2014 to 2017: a cross-sectional study. BMC Public Health. 2019 Apr 11;19(1):392.

32. Wolmarans CT, de Kock KN. The influence of health education on the prevalence, intensity and morbidity of Schistosoma haematobium infections in children over a two-year period in the Limpopo Province, South Africa. South Afr J Epidemiol Infect. 2009 Jan 1;24(1):13–7.

33. Yuan L, Manderson L, Tempongko MSB, Wei W, Aiguo P. The impact of educational videotapes on water contact behaviour of primary school students in the Dongting Lakes region, China. Trop Med Int Health. 2000;5(8):538–44.
